# A coherent transcriptional feed-forward motif model for mediating auxin-sensitive *PIN3* expression during lateral root development

**DOI:** 10.1038/ncomms9821

**Published:** 2015-11-18

**Authors:** Qian Chen, Yang Liu, Steven Maere, Eunkyoung Lee, Gert Van Isterdael, Zidian Xie, Wei Xuan, Jessica Lucas, Valya Vassileva, Saeko Kitakura, Peter Marhavý, Krzysztof Wabnik, Niko Geldner, Eva Benková, Jie Le, Hidehiro Fukaki, Erich Grotewold, Chuanyou Li, Jiří Friml, Fred Sack, Tom Beeckman, Steffen Vanneste

**Affiliations:** 1 Department of Plant Systems Biology, VIB, Ghent 9052, Belgium; 2 Department of Plant Biotechnology and Bioinformatics, Ghent University, Ghent 9052, Belgium; 3 State Key Laboratory of Plant Genomics, National Centre for Plant Gene Research, Institute of Genetics and Developmental Biology, Chinese Academy of Sciences, Beijing 100101, China; 4 Biotechnology Research Institute, Chinese Academy of Agricultural Sciences, Beijing 100081, China; 5 Department of Botany, University of British Columbia, Vancouver, British Columbia V6T 1Z4, Canada; 6 Center for Applied Plant Sciences (CAPS) and Department of Molecular Genetics, The Ohio State University, Columbus, Ohio 43210, USA; 7 Institute of Plant Physiology and Genetics, Bulgarian Academy of Sciences, Academik Georgi Bonchev Street, Sofia 1113, Bulgaria; 8 Department of Biological Sciences, Graduate School of Science, Osaka University, Osaka 560-0043 Japan; 9 Institute of Science and Technology Austria, Klosterneuburg 3400, Austria; 10 Department of Plant Molecular Biology, UNIL-Sorge, University of Lausanne, Lausanne 1015, Switzerland; 11 Key Laboratory of Plant Molecular Physiology, Institute of Botany, Chinese Academy of Sciences, Beijing 100093, China; 12 Department of Biology, Graduate School of Science, Kobe University, 1-1 Rokkodai, Kobe 657-8501, Japan

## Abstract

Multiple plant developmental processes, such as lateral root development, depend on auxin distribution patterns that are in part generated by the PIN-formed family of auxin-efflux transporters. Here we propose that AUXIN RESPONSE FACTOR7 (ARF7) and the ARF7-regulated FOUR LIPS/MYB124 (FLP) transcription factors jointly form a coherent feed-forward motif that mediates the auxin-responsive *PIN3* transcription *in planta* to steer the early steps of lateral root formation. This regulatory mechanism might endow the *PIN3* circuitry with a temporal ‘memory' of auxin stimuli, potentially maintaining and enhancing the robustness of the auxin flux directionality during lateral root development. The cooperative action between canonical auxin signalling and other transcription factors might constitute a general mechanism by which transcriptional auxin-sensitivity can be regulated at a tissue-specific level.

## Abstract (web summary)

Lateral root development is dependent on precise control of the distribution of the plant hormone auxin. Here Chen *et al*. propose the transcription factors ARF7 and FLP participate in a feed forward motif to mediate expression of the auxin transporter *PIN3* and consequently regulate lateral root development.

## Graphical abstract

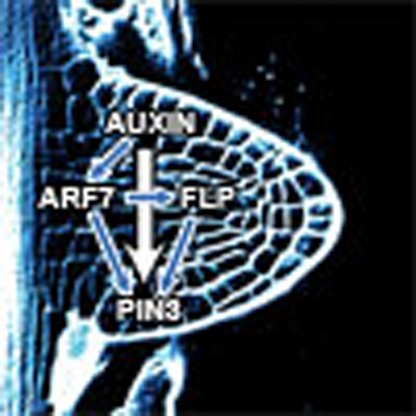

Plant growth involves the reiterative formation of post-embryonic organs such as lateral roots (LRs), tailoring the root architecture to environmentally imposed limitations. Typically, LRs are regularly spaced along the main root. The mechanism that controls this regular spacing involves oscillatory gene expression in the elongation zone of the primary root meristem^1^, also called the root clock^2^. This mechanism defines groups of cells that are competent to form LR primordia (prebranch sites). Xylem pole pericycle cells within such prebranch sites can become LR founder cells (FCs) that subsequently can be activated to initiate a LR primordium^3^. Throughout subsequent developmental stages, the nascent LR primordium communicates with the overlaying root tissues to facilitate LR emergence^4^,^5^,^6^,^7^.

The plant hormone auxin commonly plays a master role in organogenesis and morphogenesis^8^, as is explicit for LR development^9^. In the lateral root cap, the auxin precursor indole-3-butyric acid is converted to indole-3-acetic acid (IAA), feeding auxin into the root clock, thereby stimulating prebranch site formation^10^. Auxin accumulation drives the acquisition of FC identity in pericycle cells^11^, and the subsequent transition from FC to LR initiation requires stabilization of auxin signalling in these cells^12^. Subsequent LR patterning and morphogenesis are orchestrated by dynamic auxin flows^6^,^13^,^14^ as well as by complex interactions with surrounding tissues^4^,^5^,^7^,^15^.

Auxin transporters of the PIN-formed (PIN) family^16^ contribute to nearly every step of these regulatory auxin flows^6^,^12^,^13^. Prominently among the LR-regulating PINs is PIN3, which is transiently expressed in the endodermis cells overlaying FCs to promote their transition to LR initiation^12^, and is later in development also expressed in LR primordium-overlaying tissues to facilitate LR emergence^6^. These examples demonstrate the importance of dynamic *PIN* expression patterns in LR development.

The canonical auxin signalling pathway has been implicated as the main mechanism for regulating *PIN* expression^6^,^17^. This pathway is defined by the auxin-induced proteolysis of transcriptional repressor of the Aux/IAA family, thereby derepressing auxin response transcription factors (ARFs)^8^. Expression of different combinations of members of both protein families within a single cell is believed to define different auxin responsive transcriptional outputs^18^,^19^,^20^. Detailed analyses of dynamic transcriptional changes associated with auxin-induced and gravity-induced LR formation start revealing the underlying gene regulatory networks^21^. Besides the canonical auxin signalling pathway, several other transcription factors have been implicated in transcriptional control over *PIN*s; the MADS-box XAANTAL2 (XAL2/AGL14) and INDETERMINATE DOMAIN (IDD) (IDD14, IDD15, IDD16) transcription factors were recently identified as direct regulators of *PIN1* and/or *PIN4* through direct binding to the respective promoters^22^,^23^. However, almost nothing is known about how such regulators bind to *cis*-elements in the *PIN* regulatory regions and how they are integrated with the canonical auxin signalling pathway to regulate *PIN* expression during organogenetic processes such as LR development.

We previously demonstrated that *PIN3* shows remarkably dynamic expression during the patterning of stomatal complexes in the leaf epidermis^24^. We thus investigated whether the mechanisms controlling *PIN3* expression are similar during stomatal and LR development. Two genes involved in the late steps of stomatal development encode the transcription factors FOUR LIPS (FLP)/MYB124 and its closest paralogue MYB88 (ref. 25). FLP and MYB88 act redundantly to restrict guard mother cell proliferation, in part by repressing the expression of cell cycle genes^26^,^27^,^28^. Here, we propose that *FLP* transcription is auxin responsive, downstream of ARF7, and that FLP binds directly to the *PIN3* promoter as part of the molecular mechanism that controls its auxin-sensitive expression. Together, FLP and ARF7 could define a coherent feed-forward motif that controls *PIN3* expression. Mathematical modelling reveals that such transcriptional circuit could generate a temporal ‘memory' of auxin stimuli, potentially enhancing the robustness of the auxin flux patterns. Consistently, the corresponding *cis*-regulatory modules in the *PIN3* promoter are required for normal auxin-responsive *PIN3-YFP* expression that can complement the LR defect in *pin3* mutants. The feed-forward transcriptional circuit that we propose for auxin-sensitive *PIN3* transcription thus seems critical for *PIN3*-controlled LR development.

## Results

### *FOUR LIPS* is a direct target of ARF7

**Figure 1:**
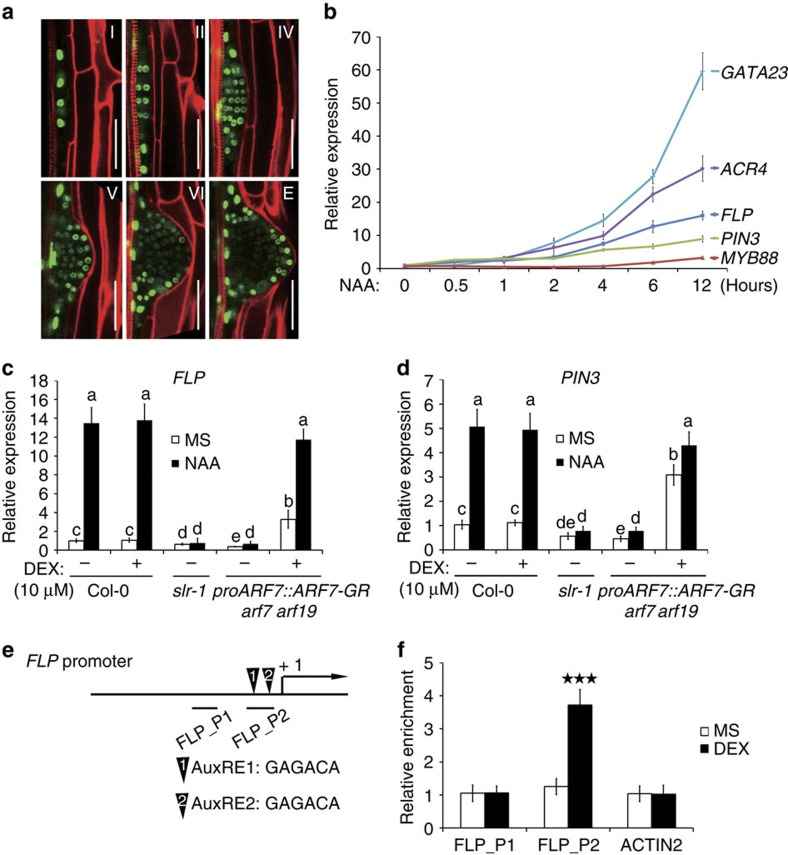
*FLP* is a direct target of ARF7. (**a**) *proFLP::NLS-GFP* expression in different stages of LR development. Scale bar, 50 μm. (**b**) qRT-PCR analysis of *GATA23*, *ACR4, PIN3*, *FLP* and *MYB88* expression during a 12 h auxin (10 μM NAA) time course from roots of 6-day-old WT seedlings. Expression levels were normalized to those of *ACTIN2*. (**c**,**d**) Relative auxin-inducibility (6 h, 10 μM NAA) of (**c**) *FLP* and (**d**) *PIN3* in WT (±10 μM DEX for 6 h), *slr-1*, and *proARF7::ARF7-GR/arf7 arf19* (±10 μM DEX for 6 h). Samples with different letters are significantly different: *P* <0.05 (Fisher's LSD mean separation test). (**e**) Schematic presentation of the *FLP* promoter (3.0 kb upstream of the translational start site at position (+1) with indication of the regions targeted for ChIP analysis (FLP_P1 and FLP_P2), and the presence of auxin response elements (AuxREs, black triangles) (**f**) Enrichment of the indicated DNA fragments (FLP_P1, and FLP_P2) following ChIP using anti-GR antibodies with *proARF7::ARF7-GR/arf7 arf19* treated for 6 h with or without DEX. A fragment from the *ACTIN2* promoter was tested as a negative control. *n*=3. Data are means ± s.d. ***P<0.001 (Student's *t*-test).

While FLP is best known for its role in the late stages of stomatal development^25^, we observed prominent *FLP* promoter activity in developing LR primordia (Fig. 1a; Supplementary Fig. 1a). Given the importance of auxin in triggering LR development, we probed whether *FLP* expression is induced by auxin. In auxin-treated roots, transcripts of *PIN3*, as well as the expression of *GATA23* and *ACR4*, which are hallmarks of LR initiation^29^,^30^, were rapidly upregulated (Fig. 1b). Similarly, transcripts of *FLP*, but not of *MYB88*, were also strongly auxin responsive (Fig. 1b). This auxin-activated *FLP* expression can be found in xylem pole pericycle cells, the cell type where LRs originate from (Supplementary Fig. 1b). The auxin signalling pathway that is central to LR induction depends largely upon the presence of the Auxin Response transcription Factor7 (ARF7) and SOLITARY ROOT/IAA14 (SLR)^9^. Together, these proteins define a canonical auxin pathway that regulates transcriptional changes associated with auxin-induced LR initiation. High auxin levels activate ARF7 transcriptional activity by destabilizing its interaction partner SLR/IAA14 (ref. 31). A gain-of-function mutation in *SLR/IAA14* (*slr-1*), or loss-of-function mutations in the *ARF7* and *ARF19* genes disrupt auxin signalling to the extent that the respective (primary) roots are completely devoid of LRs^32^,^33^. We then tested whether the auxin inducible expression of *FLP* depends upon SLR/IAA14-ARF7-based auxin signalling. In *slr-1* and in *proARF7::ARF7-GR/arf7 arf19* mutants, *FLP* expression was reduced and almost completely insensitive to auxin treatment (Fig. 1c). Moreover, when ARF7 functionality was restored to *proARF7::ARF7-GR/arf7 arf19* via dexamethasone (DEX/NAA co-treatment), *FLP* transcription regained auxin-responsiveness. A nearly identical expression profile was found for *PIN3* (Fig. 1d), which suggests it represents a direct target of this auxin signalling pathway^6^,^17^. *FLP* contains two canonical AuxREs in the 5′ region upstream of its start codon (included in FLP_P2 amplicon) (Fig. 1e). Together with its early, SLR/IAA14-ARF7-dependent expression, we hypothesized that *FLP* could be a direct target of ARF7. Therefore, we performed ChIP analyses using anti-GR antibodies on *proARF7::ARF7-GR/arf7 arf19*. In the absence of DEX, none of the fragments were significantly enriched (Fig. 1f). By contrast, the FLP_P2 amplicon became significantly enriched when plants were treated with DEX, while the enrichment of neither FLP_P1 nor ACTIN2 changed significantly (Fig. 1f). Together these data demonstrate that *FLP* is a direct target of ARF7.

### A role for *FLP* and *MYB88* in PIN3-regulated LR development

**Figure 2:**
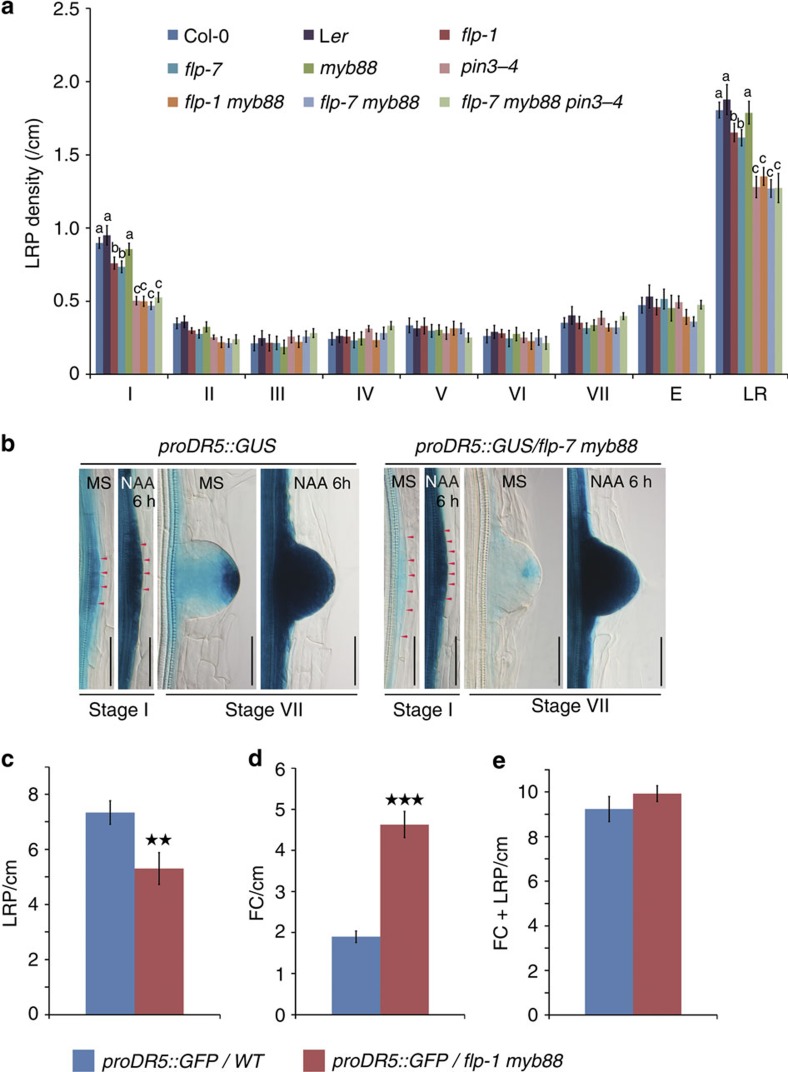
Lateral root phenotypic analysis. (**a**) Densities of the different LR stages in 6-day-old seedlings of Col-0, L*er*, *flp-1*, *flp-7*, *myb88*, *pin3-4*, *flp-1 myb88*, *flp-7 myb88* and *flp-7 myb88 pin3-4*. E=just emerged, not yet mature LRs; LR=mature LRs. Data shown are means ± s.d. of at least three independent experiments, each time sampling (*n*≥20). Samples in each stage with different letters are significantly different: *P* <0.05 (Fisher's LSD mean separation test). (**b**) Expression pattern of the auxin response output reporter *proDR5::GUS* in different LR stages of WT and *flp-7 myb88* (±10 μM NAA for 6 h). Scale bar, 50 μm. (**c**–**e**) Densities of (**c**) initiated LR primordia, (**d**) FCs and (**e**) the sum of FCs and initiated LR primordia in WT and *flp-1 myb88* as visualized by *proDR5::GFP*. Data shown are means ± s.e.m. and are representative of at least three independent experiments. (For each experiment, *n*=10). **P<0.01 and ***P<0.001 (Student's *t*-test).

To evaluate the relative contributions of *FLP* and *MYB88* expression to LR development, we analysed the LR phenotypes of corresponding single and double mutants (Fig. 2a; Supplementary Fig. 2a,b). Although *myb88* single mutants showed a wild-type (WT) root phenotype, the *flp-1* and *flp-7* alleles each exhibited reduced numbers of LRs, including a reduction in the earliest developmental stages. These phenotypes were more pronounced in the *flp-1 myb88* and in the *flp-7 myb88* double mutants. Our results thus reveal a key role for FLP in LR initiation, with MYB88 playing only a minor role, findings that echo the absence of obvious stomatal defects in the single *myb88* mutants^25^, and the lack of persistent *MYB88* expression in LR primordia (Supplementary Fig. 1c).

Given FLPs function in restricting proliferation in the stomatal cell lineage, and its necessity for cell proliferation during early LR development, we analysed the genetic interaction between *flp-7 myb88*, and a triple mutant in A2-type cyclins (*cyca2;234*) at the level of LR formation (Supplementary Fig. 3). Consistent with the inherent proliferation requirement for LR formation, the cell proliferation-impaired *cyca2;234* mutants^26^ exhibited a lower LR density than the WT, mostly resulting from a reduced level of LR initiation, a defect comparable to that of *flp-7 myb88*. Strikingly, the quintuple mutant *flp-7 myb88 cyca2;234* developed much less LR initiation sites compared to *cyca2;234* or *flp-7 myb88* alone, consistent with LR initiation defects in *cyca2;234* and *flp-7 myb88* arising via distinct pathways, suggesting that the *flp-7 myb88* LR defect is at least in part independent of the misregulated proliferation seen in *cyca2;234*.

Next, we addressed whether perturbation of LR development resulted from impaired auxin signalling. Therefore, we introgressed the synthetic auxin-response output reporter *proDR5::GUS* into *flp-7 myb88* (Fig. 2b). Whereas normally this reporter is prominently expressed in developing LRs, the *proDR5::GUS* signal was only weakly detectable in LR primordia of *flp-7 myb88* double mutants (Fig. 2b). Because this reduced *proDR5::GUS* signal might have resulted from altered auxin signalling and/or distribution, we analysed the auxin responsiveness of *proDR5::GUS* in WT and *flp-7 myb88*. A 6h auxin treatment equalized the GUS expression in both the WT and in *flp-7 myb88* (Fig. 2b). Moreover, both genotypes showed near-identical auxin-induced increases of *IAA19*, *GATA23* and *ACR4* transcripts (Supplementary Fig. 4). These results argue against the idea that the LR defect in *flp myb88* double mutants is due to a general reduction in auxin sensitivity. Instead, the altered *proDR5::GUS* expression level might be the result of abnormal auxin distribution in *flp myb88* double mutants.

To further dissect the contribution of defects in auxin transport to the LR phenotype in *flp myb88*, we focussed on the earliest visible stages of LR formation; namely LR FCs (undivided pericycle cells that exhibit *proDR5::GFP* expression)^11^ and LR initiation sites (FC that have undergone asymmetric cell division). Consistent with our other analyses (Fig. 2a), the densities of initiated LRPs were reduced in *flp-1 myb88* compared with the WT (Fig. 2c). In contrast, the FC densities in *flp-1 myb88* had dramatically increased compared to WT (Fig. 2d), resulting in cumulated densities of FCs and initiated LRPs together that were comparable to WT (Fig. 2e). An identical LR defect with less initiated LRPs but more FCs was recently also described for *pin3-4* (ref. 12). This striking resemblance prompted us to test for genetic interactions between *flp-7 myb88* and *pin3-4*. Analysis of different stages of LR development confirmed that *pin3-4* and *flp-7 myb88* LR defects were highly similar, with the most prominent defects appearing during initiation (Fig. 2a; Supplementary Fig. 2). Importantly, the LR phenotype in the *flp-7 myb88 pin3-4* triple mutants showed near-identical reductions in LR initiation as the parental mutants. The absence of clear additive effects suggests that FLP, MYB88 and PIN3 act collectively in regulating LR initiation.

### *PIN3* expression depends on FLP

**Figure 3:**
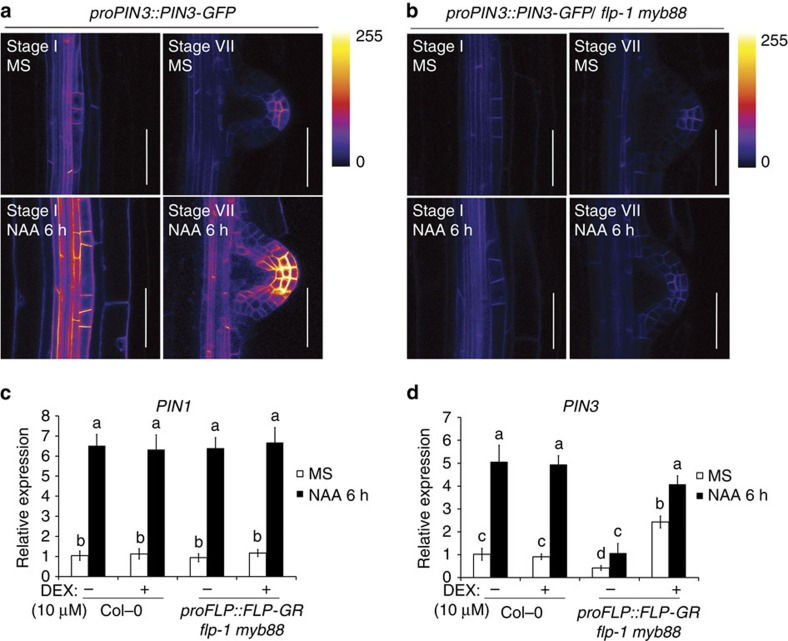
FLP controls *PIN3* expression. (**a**,**b**) Expression pattern of *proPIN3::PIN3-GFP* in (**a**) WT and (**b**) *flp-1 myb88* in early stage (LR initiation, left panel) and later stage (stage VII, right panel) of LR with or without 6 h auxin treatments (10 μM NAA). Images were taken using identical confocal settings for all samples. Scale bar, 50 μm. Relative intensities are colour coded. (**c**,**d**) Relative auxin-inducible expression (6 h of 10 μM NAA) of (**c**) *PIN1* and (**d**) *PIN3* determined by qRT-PCR from roots of WT and *proFLP::FLP-GR*/*flp-1 myb88* seedlings (± 10 μM DEX for 6 h). Data shown are means±s.d. and are representative of at least three independent experiments. Samples with different letters are significantly different: *P* <0.05 (Fisher's LSD mean separation test).

Since the expression of both *FLP* and *PIN3* are strongly auxin-inducible (Fig. 1b) and downstream of a common auxin signalling module (Fig. 1c,d), we tested whether auxin-induced *PIN3* expression requires functional FLP protein. Indeed, *proPIN3::PIN3-GFP* showed reduced auxin-inducible expression in LR primordia when introgressed into *flp-1 myb88* (Fig. 3a,b). In addition, we analysed *PIN3* expression in *proFLP::FLP-GR/flp-1 myb88* lines in which the LR defect, but not the stomatal phenotype, could be completely complemented by DEX-treatment (Supplementary Fig. 5). In this line, *PIN1* expression levels exhibited auxin responsive amplitudes that were comparable to those of the WT, independently of DEX treatment (Fig. 3c). By contrast, in the absence of DEX, the auxin inducibility of *PIN3* in roots was severely reduced (Fig. 3d). Importantly, DEX-treatment restored the auxin responsiveness of *PIN3* to WT levels in a *proFLP::FLP-GR/flp-1 myb88* background (Fig. 3d). These results show that FLP is required for auxin-induced *PIN3* expression, as was also shown for *PIN3* expression in the columella of the primary root meristem^34^.

### *PIN3* is directly regulated by FLP and ARF7

**Figure 4:**
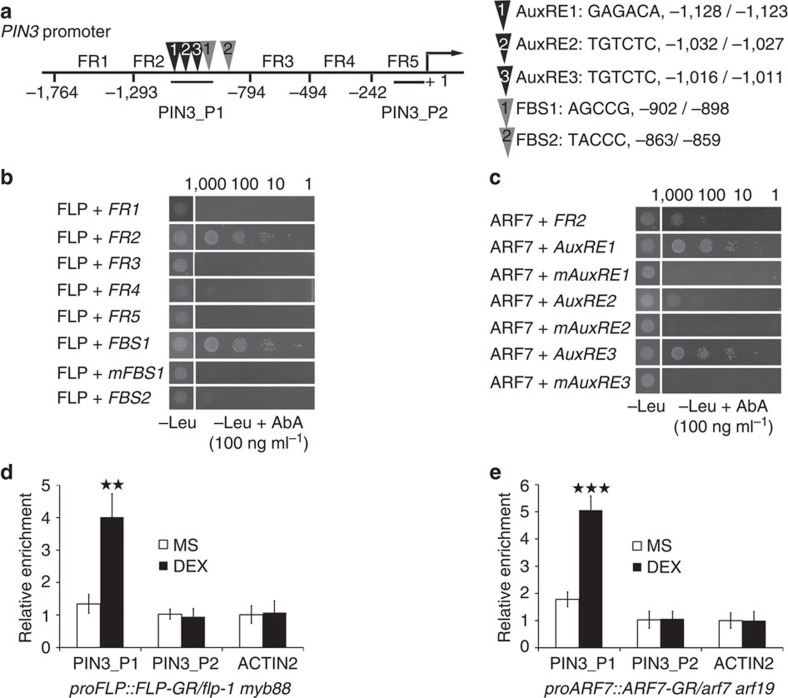
FLP and ARF7 are direct regulators of *PIN3* expression. (**a**) Schematic presentation of the *PIN3* promoter (1.8 kb upstream of the translational start site at position (+1) with indication of the regions targeted for ChIP analysis (PIN3_P1 and PIN3_P2), the fragments used for yeast-one-hybrid (FR1-5) and the presence of auxin response elements (AuxREs, black triangles) and FLP binding sites (FBS, grey triangles) in fragment FR2. (**b, c**) Yeast-one-hybrid analysis of the interaction of (**b**) FLP with *PIN3* promoter fragments (FR1-5), FBS1, a mutated FBS1 (mFBS1) and FBS2, and (**c**) of ARF7 with *PIN3* promoter fragment FR2, its internal AuxREs (AuxRE1-3) and corresponding mutated versions (mAuxRE1-3). Left is yeast grown on SD–Leu medium in absence of Aureobasidin A (AbA). Right are the corresponding yeast strains in a dilution series grown on SD–Leu medium in the presence of 100 ng ml^−1^ AbA, a concentration where no autoactivation was detected for any of the strains. (**d**,**e**) Enrichment of the indicated DNA fragments (PIN3_P1, and PIN3_P2) following ChIP using anti-GR antibodies with (**d**) *proFLP::FLP-GR/flp-1 myb88* or (**e**) *proARF7::ARF7-GR/arf7 arf19* treated for 6 h with or without DEX. A fragment from the *ACTIN2* promoter was tested as a negative control. Data are presented as means ± s.d. **P<0.01, ***P<0.001 (Student's *t*-test). The experiment was repeated three times with similar results.

Because both the expression level of *FLP* and *PIN3* are relatively rapidly induced by auxin and depend upon SLR/IAA14-ARF7 signalling, we explored whether *FLP* might be a downstream effector of the auxin signalling pathway by directly regulating *PIN3* expression. Five different promoter fragments of the *PIN3* promoter (FR1-5) were tested for their ability to interact with FLP via yeast-one-hybrid assays (Fig. 4a). Only the fragment FR2 was found to generate Aureobasidin A (AbA)-resistant yeast growth, suggesting that this fragment can recruit FLP (Fig. 4b). This fragment contains two sites (FBS1=AGCCG, FBS2=TACCC) that meet the experimentally determined [A/T/G][A/T/G]C[C/G][C/G] consensus sequence for FLP binding^27^. Using short fragments containing either FBS, only the construct containing FBS1 could render the yeast resistant to AbA (Fig. 4b). Moreover, no such AbA-resistance was found when the FBS1 sequence was changed to AATTA (mFBS1) within this construct. These data suggest that FLP can bind directly to the *PIN3* promoter via FBS1.

We then tested whether this interaction also occurs *in planta* using ChIP-PCR analysis with anti-GR antibodies (Fig. 4d). FLP activity was controlled conditionally in the *proFLP::FLP-GR/flp-1 myb88* line, and analysed for a differential enrichment at two different regions in the *PIN3* promoter (amplicons PIN3_P1 and PIN3_P2) (Fig. 4a) as well as one in the unrelated *ACTIN2* promoter. Compared to the mock treatment, a 6-h DEX treatment strongly enriched the amplicon closest to FBS1 (PIN3_P1), whereas DEX had no impact on the enrichment of PIN3_P2 and ACTIN2 (Fig. 4d). These data further confirm that FLP is a direct regulator of *PIN3* expression, a finding that was corroborated by Wang *et al*.^34^.

However, it has been reported that auxin-inducible *PIN3* expression does not require *de novo* protein synthesis^6^,^17^, suggesting that *PIN3* is a primary auxin-responsive gene, thus not requiring FLP as an intermediate regulator. Interestingly, the *PIN3* promoter contains three ‘canonical' auxin responsive elements (AuxREs)^35^,^36^, all of which are located within FR2, in close proximity to FBS1 (Fig. 4a). Using yeast-one-hybrid assays we found that ARF7 can interact with FR2 (Fig. 4c). In addition, short fragments spanning the individual AuxREs, also interacted with ARF7, and these interactions were lost when the AuxRE sequences were mutated (mAuxRE). We then tested whether ARF7 interacts with the *PIN3* promoter *in planta* via ChIP-PCR in *proARF7::ARF7-GR/arf7 arf19*. In the absence of DEX, PIN3_P1 was only mildly enriched compared with PIN3_P2 and ACTIN2 (Fig. 4e). By contrast, the PIN3_P1 amplicon became strongly enriched when plants were treated with DEX, but DEX treatment did not stimulate the enrichment of PIN3_P2 and ACTIN2, confirming that *PIN3* is indeed a primary auxin responsive gene. Thus our data demonstrate that *PIN3* is a direct target of both ARF7 and FLP, two transcription factors that are required for auxin-responsive *PIN3* transcription.

### PIN3-driven LR development requires ARF7 and FLP binding

**Figure 5:**
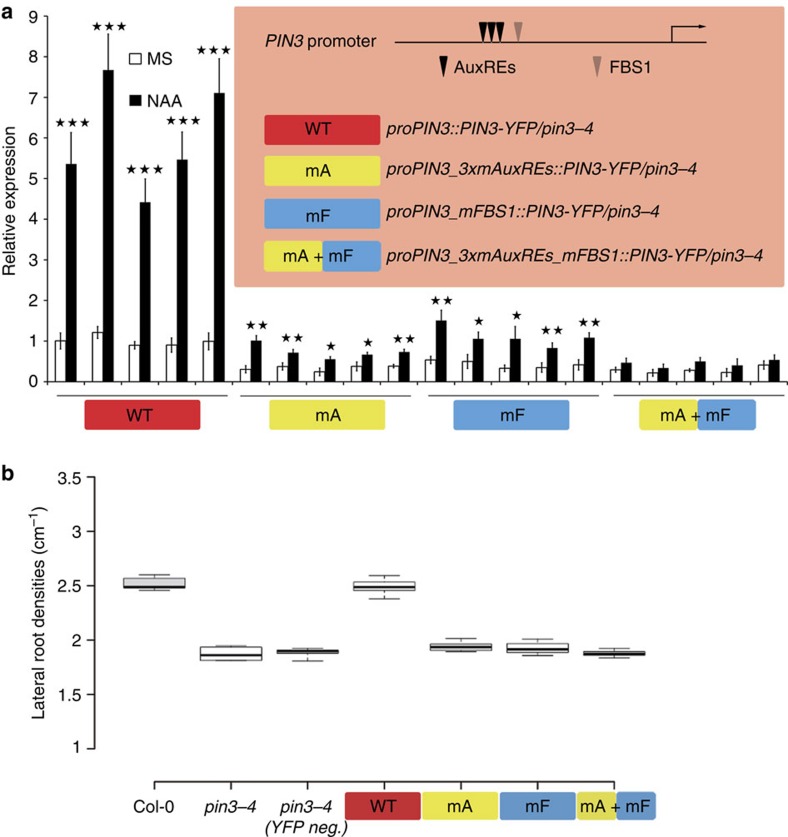
*In planta* assessment of the relevance of ARF7 and FLP recruitment on auxin-inducibility of *PIN3* and PIN3-dependent LR development. (**a**) Relative auxin-inducibility (10 μM NAA for 6 h) of *PIN3-YFP* driven by the indicated *proPIN3* variants. Roots of YFP positive plants were used for qRT-PCR assay. Scheme of *PIN3* promoter illustrating by colour-coding the variants of the *PIN3* constructs used to complement the *pin3-4* mutant. Data are means ± s.d. of at least three independent experiments. Asterisks denote Student's *t*-test significance: *P<0.05, **P<0.01 and ***P<0.001. (**b**) Quantification of the LR densities in 8-day-old seedlings of Col-0, *pin3-4*, YFP-negatives segregating among transgenic plants (*proPIN3_WT*), *proPIN3_WT*, *proPIN3_mA*, *proPIN3_mF* and *proPIN3_mA+mF*. Box plot description: centre lines show the medians of the averages of five independent transgenic lines, calculated for at least 20 individual plants per line; box limits indicate the 25th and 75th percentiles as determined by R software; whiskers extend to minimum and maximum values. *n*=5.

Next, we evaluated the role of FLP- and/or ARF7-mediated regulation of *PIN3* on LR development. For this purpose, we designed *proPIN3* variants of 1.8 kb, containing specific mutations predicted to abrogate activation by FLP (mF) or by ARF7 (mA), then fused them to *PIN3-YFP* and transformed them into *pin3-4* (Fig. 5). Five independent lines were selected per construct, to minimize user-biased pre-selection for transgenic lines, and YFP positive individuals were analysed for auxin-responsive *PIN3-YFP* amplitude and LR density. In lines expressing *PIN3-YFP* from the reference *PIN3* promoter (WT) a 5–8-fold higher *PIN3-YFP* expression was measured after auxin treatment, an amplitude within the order of magnitude of what is typically observed for endogenous *PIN3* (Fig. 5a). Although strongly reduced, the *proPIN3::PIN3-YFP* variants impaired in either FLP or ARF7 recruitment (mF or mA) still showed significant auxin responsive activity. When recruitment of both FLP and ARF7 was impaired (mF + mA), auxin could no longer up-regulate *PIN3-YFP* expression significantly. These results are consistent with a model where FLP and ARF7 constitute the core mechanism by which auxin activates *PIN3* expression.

These lines thus allowed us to probe the functional importance of FLP- and/or ARF7- regulated *PIN3* expression for LR development. Therefore, we analysed the ability of these constructs to complement the LR defect in *pin3-4*. When *PIN3-YFP* was expressed under the control of the reference *PIN3* promoter (WT), the LR density was restored to WT (Col-0) levels, whereas segregating YFP negative plants still showed the *pin3-4* LR defect (Fig. 5b). In contrast, none of the mutant *PIN3* promoter constructs were able to rescue the *pin3-4* LR defect. Importantly, while mutations in either FLP or ARF7 binding sites still showed some *PIN3-YFP* auxin-mediated upregulation, this auxin-responsive *PIN3-YFP* expression was not sufficient to complement the LR defect in *pin3-4*. These results demonstrate that the regulation of auxin-sensitive *PIN3* expression as defined by FLP as well as ARF7 is a crucial parameter for LR development.

### Model of ARF7- and FLP-dependent *PIN3* transcription

**Figure 6:**
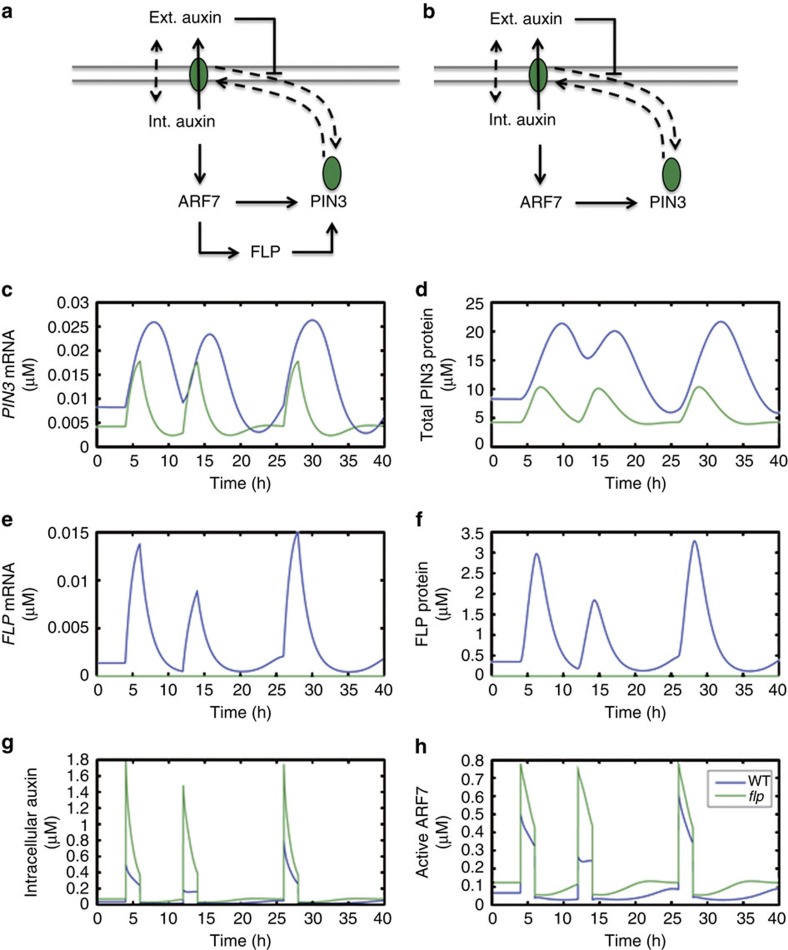
Simulation of ARF7 and FLP-regulated *PIN3* dynamics. (**a**) The coherent feed-forward motif (FFM) scheme and (**b**) the no-FFM scheme used to model auxin-induced *PIN3* transcription. (**c**–**h**) Simulations of a PIN3-dependent auxin transport model subject to extracellular auxin pulses. Simulations were performed on model systems with (blue) and without (green) a transcriptional FFM involving FLP (see Methods), using the parameter settings given in Supplementary Table 1. Two-hour long 1 μM auxin pulses were given to the system at *t*=4 h*, t*=12 h and *t*=26 h. The subplots depict the resulting dynamics of *PIN3* mRNA (**c**), total PIN3 protein, that is, the sum of intracellular and membrane-bound PIN3 (**d**), *FLP* mRNA (**e**), FLP protein (**f**), intracellular auxin (**g**) and active ARF7 (**h**) concentrations over time. The model simulations indicate that the FLP feed-forward circuit causes prolonged transcriptional activation of *PIN3*, resulting in an enhanced memory effect by which auxin stimulation reduces the impact of subsequent extracellular auxin stimuli on ARF7 activity and downstream signalling for a prolonged period of time. The system dynamics are qualitatively independent of the exact parameter settings used (see Supplementary Figs 6-10).

To probe how the joint activity of ARF7 and FLP impacts on auxin-regulated *PIN3* transcription, we developed a mathematical model that integrates ARF7 and FLP into a coherent feed-forward motif (FFM) regulating *PIN3* transcription and PIN3-mediated auxin transport (Fig. 6a). In parallel, we tested a model that lacks FLP function (mimicking a *flp* mutation) (Fig. 6b). A full description of the model and the parameter settings used can be found in the Methods section. Our simulation results indicate that the FFM may function to prolong and amplify *PIN3* expression in response to auxin stimuli (Fig. 6c–h). In contrast to the system lacking the FFM, *PIN3* in the FFM model continues to be actively transcribed for several hours upon removal of the auxin stimulus. Such a delay in response shutdown after stimulus removal is thought to be a key feature of coherent OR-type feed-forward circuits^37^. Simulations with other parameter settings gave qualitatively similar results (see Supplementary Figs 6–9). To test the sensitivity of the observed PIN3 response delay to variations in the model parameters, we screened the ratio of the total PIN3 protein concentration response time upon auxin stimulus removal for model circuits with and without FFM as a function of single parameter changes (see Methods and Supplementary Fig. 10). For none of the parameters screened, the FFM/no FFM response time ratio drops below 1 in the parameter range profiled, indicating that PIN3 response delay upon auxin stimulus removal is a robust feature of the circuit incorporating the FLP FFM. As expected, the response time ratio approximates 1 when FFM parameters approach values that make the FFM non-functional, for example, very-low *FLP* mRNA or protein synthesis rates (*α*_FLP_ or *β*_FLP_), very-high *FLP* mRNA or protein decay rates (*γ*_FLP_ or *δ*_FLP_), or very-high half-max constants for FLP-dependent activation of *PIN3* transcription or ARF7-dependent activation of *FLP* transcription (*K*_FP_ or *K*_AF_). Long *PIN3* mRNA or protein half-lives (small *γ*_PIN3_ or *δ*_PIN3_) also appear to offset the effect of the FFM (see Supplementary Fig. 10).

In the present context, we speculate that the FFM regulation of auxin signalling provides a temporary memory of cellular auxin levels to specific specialized cells, potentially enhancing the robustness of auxin flux patterns and focussing auxin maxima during post-embryonic patterning processes, such as LR initiation and development. In addition, our simulations indicate that delayed *PIN3* down-regulation might temporarily capacitate the system for mitigating the effects of subsequent auxin stimuli on ARF7 activity and downstream auxin signalling (Fig. 6h). Together our data represent a first example of a mechanism by which plant endogenous cues such as auxin can become ‘memorized' to temporarily sustain transcriptional auxin signalling in selected cells for specific transcripts.

## Discussion

Local auxin accumulation serves an essential role in triggering transcriptional changes associated with developmental transitions during plant organogenesis, as is reflected during LR development. A remarkable feature of auxin transport orchestrating organogenesis and regeneration, is the multi-level feed-back regulation of PIN-mediated auxin transport flows that are reinforced by the hormone itself^8^. Prominently among them is the auxin-induced transcription of *PINs*^17^, stimulating the cellular auxin transport capacity. Although not studied in detail, it has been generally accepted that the auxin-sensitive expression of several *PINs*, including *PIN3*, is directly activated by the canonical auxin signalling^6^,^17^. Our data further elaborate this notion by demonstrating that direct binding of ARF7 to the *PIN3* promoter is critical for auxin-sensitive transcription of *PIN3*.

Such auxin-dependent regulation might mainly act to amplify the complex, developmentally wired *cis*-regulatory architectures that underlie the tissue-specific expression patterns of the different *PIN*s^38^. Here, we propose that the auxin-sensitive expression of *PIN3* depends on binding of both ARF7 and FOUR LIPS, a MYB transcription factor previously characterized for its role in guard cell development. In addition, we found that ARF7 also controls auxin-sensitive *FLP* expression, thus defining a coherent FFM for auxin-induced *PIN3* expression. Mathematical simulations revealed that this configuration could amplify the auxin-response output of *PIN3* transcription and also temporarily sustain elevated *PIN3* transcription after the auxin-stimulus is removed. This could allow for depletion of cellular auxin below the levels that were initially needed to activate *PIN3* transcription, and for temporal buffering of random auxin fluctuations. This enhanced auxin transport capacity seems to stimulate FCs to proceed into LR initiation. Such transcriptional control mechanisms could also be relevant for developmental processes that are associated with auxin depletion, such as fruit valve specification^21^,^39^ and stomatal development and patterning^24^. Interestingly, coherent FFMs of the type described here are abundantly found in transcriptional networks in yeast, animals and plants^40^,^41^,^42^,^43^, suggesting that this motif might represent an evolutionarily favoured solution to obtain a response delay upon stimulus shutdown.

Our data suggest that this auxin-sensing circuitry of *PIN3* expression is involved LR initiation. In this developmental process, the high auxin concentrations that promote the progression from FC to LRI depends in part on PIN3-mediated auxin transport^12^, as illustrated by the diminished DR5 expression in *flp myb88* LR primordia. Similar transcriptional architectures could underlie the auxin-sensitive expression of other *PIN*s, including *PIN1* and *PIN4* that were found to be directly controlled by the auxin-inducible MADS-box XAL2/AGL14 (ref. 22), and *PIN7* that was also found to be directly regulated by FLP binding to its promoter^34^.

## Methods

### Plant materials and growth conditions

*Arabidopsis* seeds were surface-sterilized for 15 min in 10% bleach, washed four times with sterile water, and plated on half-strength Murashige and Skoog medium (0.8% agar). Plants were stratified at 4 °C for 2 days in darkness and then transferred to a growth chamber at 22 °C under continuous illumination (light intensity 120 μmol m^−2^ s^−1^).

*Arabidopsis thaliana* ecotypes Columbia (Col-0), and L*er* were used as controls. The following lines/seeds/constructs were used in this study: *flp-1* (ref. 44), *flp-7* (ref. 25), *myb88* (SALK_068691)^25^, *flp-1 myb88* (ref. 25), *flp-7 myb88* (ref. 25), *proFLP::GUS-GFP*^25^, *cyca2;234* (ref. 26), *flp-7 myb88 cyca2;234* (ref. 26), *slr-1* (ref. 32), *proARF7::ARF7-GR/nph4-1(arf7)arf19-1* (ref. 33), *pin3-4* (SALK_038609)^45^, *proDR5::GUS*^46^, *proDR5rev::GFP*^47^ *proPIN3::PIN3-GFP*^45^. Published mutants and reporter lines were crossed into *flp-1 myb88* and *flp-7 myb88*. *proFLP::FLP-GR/flp-1 myb88*, *proFLP::NLS-GFP*, *proPIN3::PIN3-YPF/pin3-4* lines were generated through *Agrobacterium*-mediated floral dip transformation^48^.

### GUS staining and microscopy

The β-glucuronidase (GUS) assays were performed as follows: seedlings were incubated in GUS staining buffer (0.1 M Tris pH7.5 containing 2.9 mg ml^−1^ NaCl, 6.6 mg ml^−1^ K_3_Fe(CN)_6_) at 37 °C overnight^49^. For microscopic analysis, samples were cleared by mounting in lactic acid (Acros Organics, Geel, Belgium) or were transferred to 0.24 N HCl in 20% at 57 °C for 15 min. This solution was replaced with 7% NaOH, 7% hydroxylamine-HCl in 60% ethanol for 15 min at room temperature. Roots were then rehydrated for 5 min each in 40, 20 and 10% ethanol, and infiltrated for 15 min in 5% ethanol, 25% glycerol. Roots were mounted in 50% glycerol on glass microscope slides^50^. All samples were analysed using a BX53 Olympus microscope. Fluorescence images were acquired by a Zeiss 710 confocal laser scanning microscope equipped with a C-Apochromat × 40 water immersion objective. If needed for visualization purposes, further processing was done using Image J software by uniformly changing brightness and contrast to the entire image per channel. For the images in Fig. 1a, the smoothing function was applied to both channels.

### Phenotyping and statistics

To analyse stages of LR development, about 20 roots from 6-day-old seedlings were processed per data point and genotype. Plant material was cleared following the more elaborate protocol as described above^50^. Root lengths were measured with the Image J software (NIH; http://rsb.info.nih.gov/ij). LRPs were counted with a differential interference contrast microscope (BX53; Olympus). LRP density was calculated as the ratio of the total number of LRP over the sum of root length. Phenotypic analysis of FC and LRP was done as described^12^. In brief, 10 roots of 5-day-old seedling (grown at 21°C 16 h light 8 h dark) were analysed per experiment. The counting of FCs and LRP was performed in the direction from root tip towards the root base. Zeiss LSM 710 confocal microscope with a × 40 (water immersion) objective with GFP settings (excitation 488 nm, emission 507 nm) were used to identify and count *DR5rev::GFP*-positive signals in the pericycle. GFP signals accompanied with nuclear divisions were scored as LRP, whereas FCs were counted when no cell divisions were observed. The data are represented as averages of three independent experiments, unless indicated differently. The statistical significance was evaluated by Student's *t*-test analysis. For multiple comparisons, an analysis of variance followed by Fisher's Least Significant Difference (LSD) mean separation test (SPSS) was performed on the data. Samples with different letters are significantly different at *P*<0.01 or *P*<0.05. Boxplots were generated using BoxPlotR^51^.

### Cloning

To construct *proFLP::FLP-GR*, a *FLP* promoter region (±3 kb upstream of translational start) was amplified with primers proFLP-F and proFLP-R and cloned into pDONR™P4-P1R. The *FLP* cDNA was amplified with primers FLP-F and FLP-R and cloned into pDONR™221; The above-mentioned pDONR vectors were subcloned together with a Glucocorticoid ORF containing (pDONR™P2R-P3) into pB7m34GW^52^,^53^ with MultiSite Gateway Three-Fragment Vector Construction Kit (Invitrogen). cDNA of ARF7 was PCR amplified with primers ARF7-F and ARF7-R, and cloned into pDONR™221. *proFLP* in pDONR™P4-P1R was subcloned into pEX-K7SNFm14GW to construct *proFLP::NLS-GFP.*

A promoter 1.8 kb upstream of the *PIN3* translational start was cloned into pDONR™P4-P1R with primers proPIN3_FL_F and proPIN3_FL_R. Site-directed mutagenesis was performed using PCR to mutate AuxREs and FBS. The resulting promoters were cloned into pDONR™P4-P1R, and together with *PIN3-YFP* in pDONR™221 (ref. 45), subcloned into pB7m24GW^52^,^53^. The above-mentioned primers are listed in Supplementary Table 2.

### Gene Expression Analysis by qRT-PCR

For qRT-PCR analysis, 6-day-old seedlings were treated with NAA (10 μM) and/or DEX (10 μM) at different time points and roots were harvested for RNA extraction. RNA was extracted with an RNeasy kit (QIAGEN). Poly(dT) cDNA was prepared from 1 μg of total RNA with SuperScript III reverse transcriptase (Invitrogen) and analysed on a LightCycler 480 apparatus (Roche Diagnostics) with the SYBR Green I Master kit (Roche Diagnostics) according to the manufacturer's instructions. Primer pairs were designed with Beacon Designer 4.0 (Premier Biosoft International) (Supplementary Table 2). All individual reactions were performed as biological triplicates. Data was analysed with qBase^54^. Expression levels were normalized to those of *ACTIN2*. The statistical significance was evaluated by Student's *t*-test analysis for pair-wise comparisons and by Fisher's LSD means separation test (SPSS) for multiple comparisons.

### ChIP-qPCR assay

Eight-day-old seedlings of *proFLP::FLP-GR/flp-1 myb88* or *proARF7::ARF7-GR/arf7 arf19* were treated with 10 μM DEX for 6 h. One gram root material per sample was used for ChIP experiments. Plant materials were cross-linked in 1% formaldehyde and their chromatin isolated. Anti-GR antibody ab3580 (abcam) (1:500) was used to immunoprecipitate the protein-DNA complex, and the precipitated DNA was purified using a PCR purification kit (Qiagen) for qRT-PCR analysis. The ChIP experiments were performed three times. Chromatin precipitated without antibody was used as negative control, while the isolated chromatin before precipitation was used as input control^55^,^56^. The enrichment of DNA fragments was determined by quantitative PCR using the following primer pairs: proACT2_F and proACT2_R; proFLP(P1)_F, proFLP(P1)_R, proFLP(P2)_F, proFLP(P2)_R, proPIN3(P1)_F and proPIN3(P1)_R; proPIN3(P2)_F and proPIN3(P2)_R (Supplementary Table 2).

### Yeast-one hybrid

Yeast-one-hybrid assays were performed with the kit provided by Clontech (Matchmaker One-Hybrid Library Construction and Screening kit) using the Y1HGold yeast strain according to the manufacturer's instructions. This system detects binding events by resistance against the antibiotic Aureobasidin A (AbA).

The primers used for cloning the related cDNAs or promoter DNAs are listed in Supplementary Table 2. The cDNA of *ARF7* and *FLP* in pDONR™221 were subcloned into pGADT7. The promoter fragments of *PIN3* were cloned into the SacI/SalI sites of pAbAi vector, and efficiently integrated into the genome of the Y1H Gold yeast strain by homologous recombination on SD-U medium. Matchmaker™ Insert Check PCR Mix was used to verify positive colonies. Autoactivation tests for the promoter were conducted in the range from 0 to 500 ng ml^−1^ AbA, and background activation was detected up to 50 ng ml^−1^. An AbA concentration of 100 ng ml^−1^ was used to screen positive colonies. Then pGADT7 prey vectors harboring ORF of *ARF7* or *FLP* were transformed into the above-mentioned yeast strain that have already integrated the promoter fragments of *PIN3* or *FLP*. After having grown on SD-Leu for 2–3 days, the colonies able to grow on the selective medium were transferred to 100 μl of sterile ultrapure water and spotted as 5 μl droplets onto SD-Leu+AbA plates. Plates were incubated at 28 °C for 3–4 days.

### Modelling the effects of FLP on PIN3 and auxin dynamics

We mathematically modelled the impact of the FLP FFM on PIN3 expression dynamics starting from an earlier model by Jönsson *et al*.^57^. The revised model is defined by the following ordinary differential equations:


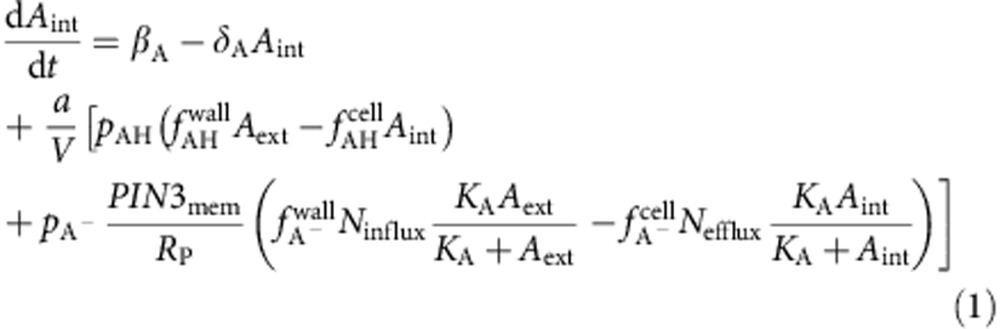



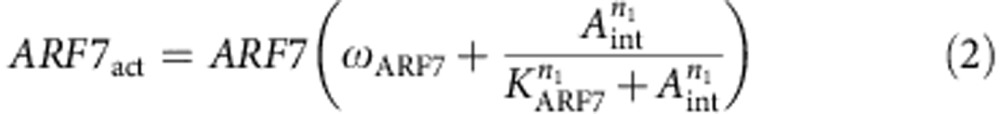



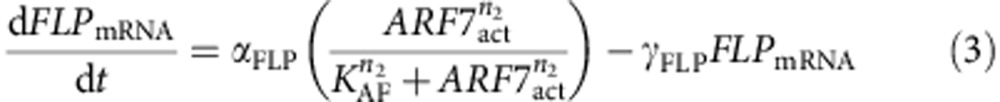



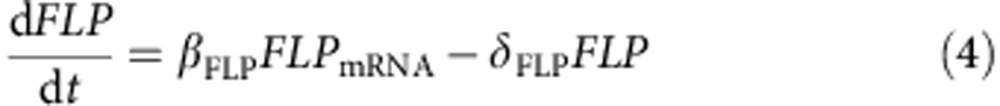



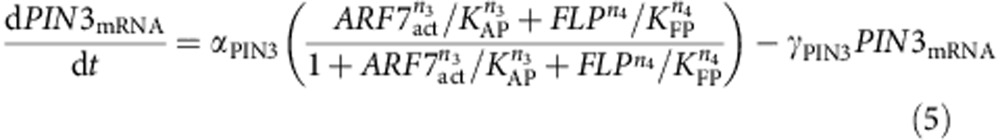



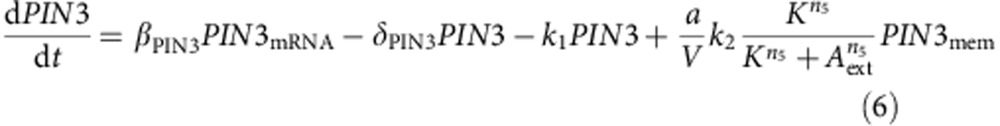



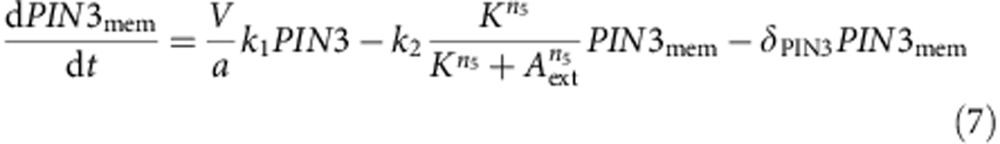


with *A*_int_ and *A*_ext_ the intra- and extracellular auxin concentrations, respectively, *ARF*7_act_ the concentration of active ARF7 protein, *FLP*_mRNA_ and *FLP* the concentrations of FLP mRNA and protein, *PIN*3_mRNA_ the concentration of PIN3 mRNA, and *PIN*3 and *PIN*3_mem_ the concentrations of intracellular and membrane-localized PIN3 protein. *α* and *β* parameters denote mRNA and protein (or auxin) production rates, respectively, and *γ* and *δ* parameters denote the corresponding degradation rates. *K*′s indicate half-maximal activation constants, and *n*′s are activation curve Hill coefficients. Detailed parameter definitions and dimensions, as well as the default parameter values used, are presented in Supplementary Table 1 (refs 57, 58, 59).

As in Jönsson *et al*.^57^, the intracellular auxin concentration in our model is influenced by intracellular auxin production and degradation, and by passive and active auxin transport across the cell membrane. In contrast to Jönsson *et al*.^57^, the auxin dynamics in the cell wall are not modelled explicitly. Instead, the temporal profile of the extracellular auxin concentration (*A*_ext_) is predefined for each simulation, in terms of a constant background concentration and auxin pulses during specific epochs (see below). In equation (1), the saturability of *PIN*3_mem_-mediated active auxin transport is modelled slightly differently from the original model^57^. In particular, *PIN*3_mem_/*R*_P_ (with *R*_P_ a reference *PIN*3_mem_ concentration) is used as a factor to modulate the membrane permeability *p*_A_^−^, instead of limiting the maximum membrane PIN concentration to 1.0 μmol per unit area as in ref. 57. In addition, the form of the auxin-dependent saturation characteristic was reverted to the form used earlier by Mitchison *et al*.^58^, which includes an additional *K*_A_ factor in the numerator of the Michaelis-Menten relation, to restore the unit balance. In equations (6) and (7), the formulation of auxin-dependent PIN3 membrane transport and PIN3 membrane localization has been changed to reflect the observation that auxin inhibits the internalization of membrane-bound PIN3 rather than stimulating PIN3 delivery to the plasma membrane^60^,^61^, and to better reflect the membrane concentration nature of *PIN*3_mem_ (μmol dm^−2^).

We complemented this auxin transport model with equations describing the dynamics of the transcriptional feed-forward circuit impacting PIN3 expression (Equations (2)–(6), 3, 4, 5, 6). Assuming a constant ARF7 protein concentration (*ARF*7=1 μM), ARF7 activity kinetics are modelled as a combination of basal, auxin independent ARF7 activity (*ω*_ARF7_) and auxin-dependent ARF7 activity (Equation (2)). Equation (3) describes how FLP mRNA levels are influenced by *ARF*7_act_-dependent transcription and mRNA degradation, while equation (4) describes FLP protein concentration changes as a function of protein production and degradation. Equation (5) incorporates the effects of active ARF7 and FLP on *PIN3* transcription, as well as an mRNA degradation term. Equation (6) incorporates PIN3 protein production and degradation terms, in addition to the membrane transport-related terms discussed above. A protein degradation term was also added to equation (7), under the assumption that membrane-bound PIN3 is also earmarked for degradation.

Simulations were performed in MATLAB R2013b, using an implicit numerical solver with adaptable time step used for solving initial value problems for stiff ordinary differential equations (ode23s.m function). The MATLAB scripts are available from http://www.psb.ugent.be/esb/ESB/FLP_FFM_SI.html. The default simulations (Fig. 6c–h) were performed using the parameter settings described in Supplementary Table 1. mRNA and protein degradation rate constants were set to reflect typical mRNA and protein half-lives of ∼1 h. In the default settings, FLP was set to have a shorter protein half-life (30 min) than PIN3 (2 h) and the half-max constants for FLP- and ARF7-dependent activation of *PIN3* transcription (*K*_FP_ and *K*_AP_) were set to 0.6 and 0.3 μM, respectively, in order to achieve a ∼50% drop in PIN3 expression level as observed in the *flp* mutant after 6 h (see Fig. 3d). Initial auxin, PIN3 and FLP levels were set to the steady-state levels obtained for a constant extracellular auxin concentration (*A*_ext_) of 0.1 μM. Simulations of the *flp* mutant were performed by setting *α*_FLP_, *β*_FLP_, *γ*_FLP_, *δ*_FLP_ and the initial FLP mRNA and protein concentrations to zero. In all simulations, a background extracellular auxin concentration of 0.1 μM was assumed, and 2 h-long 1 μM extracellular auxin pulses were simulated at *t*=4–6 h, *t*=12–14 h and *t*=26–28 h.

### PIN3 response delay upon stimulus removal to parameters

Although the parameters listed in Supplementary Table 1 are within biologically reasonable ranges, the resulting model cannot be taken to be quantitatively accurate, as this would require quantitative measurements of all model parameters and absolute molecular concentration ranges *in vivo*. To test the robustness of our modelling-based conclusions, we performed a series of simulations screening the effects of parameter changes on the qualitative dynamics of the FFM versus no-FFM circuits. In each simulation, one parameter was changed from its default value listed in Supplementary Table 1, while the other parameters were kept at their default values. Hill coefficients *n*_1_, *n*_2_, *n*_3_, *n*_4_, *n*_5_ were screened in the range [1, 3], *ω*_ARF7_ was screened in the range [0, 1] and the other parameters were screened in a range encompassing one order of magnitude above and below the default values listed in Supplementary Table 1 (with the exception of *β*_A_, the midrange value of which (on log scale) was set to 5 × 10^−6^ s^−1^ instead of 0 s^−1^ for screening purposes). The resulting alternatively parameterized circuits were simulated with and without FFM under a 1 μM extracellular auxin stimulus until steady-state was reached, after which the auxin stimulus was shut down and the PIN3 protein response time was measured, defined as the time it takes the system to bridge half of the PIN3 protein concentration difference between the auxin-stimulated and unstimulated steady states. FFM/no FFM response time ratios >1 indicate that the circuit with FFM exhibits a delayed PIN3 protein response upon auxin stimulus shutdown.

## Additional information

**How to cite this article:** Chen, Q. *et al*. A coherent transcriptional feed-forward motif model for mediating auxin-sensitive *PIN3* expression during lateral root development. *Nat. Commun.* 6:8821 doi: 10.1038/ncomms9821 (2015).

## Supplementary Material

Supplementary Information Supplementary Figures 1-10, Supplementary Tables 1-2 and Supplementary References
