# Supplementary material for: A coherent transcriptional feed-forward motif model for mediating auxin-sensitive PIN3 expression during lateral root development
Source: Nat Commun. 2015 Nov 18;6:8821. doi: 10.1038/ncomms9821 (PMC4673502; doi:10.1038/ncomms9821)
Supplement: Supplementary Information — Supplementary Figures 1-10, Supplementary Tables 1-2 and Supplementary References [file ncomms9821-s1.pdf]

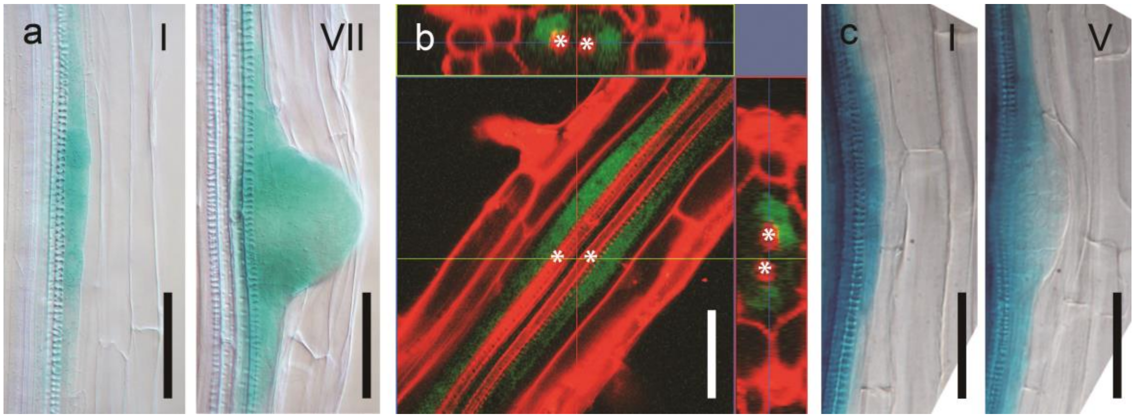

Supplementary Figure 1 (**a**) Expression pattern of *proFLP::GUS-GFP* in stage I and VII of LR development. (**b**) Z-stack analysis of *proFLP::GUS-GFP* treated with 10µM NAA for 6h. Asteriks indicate xylem strands. (**c**) Expression pattern of *proMYB88::GUS-GFP* in stage I and V of LR development. Scale bar = 50 µm

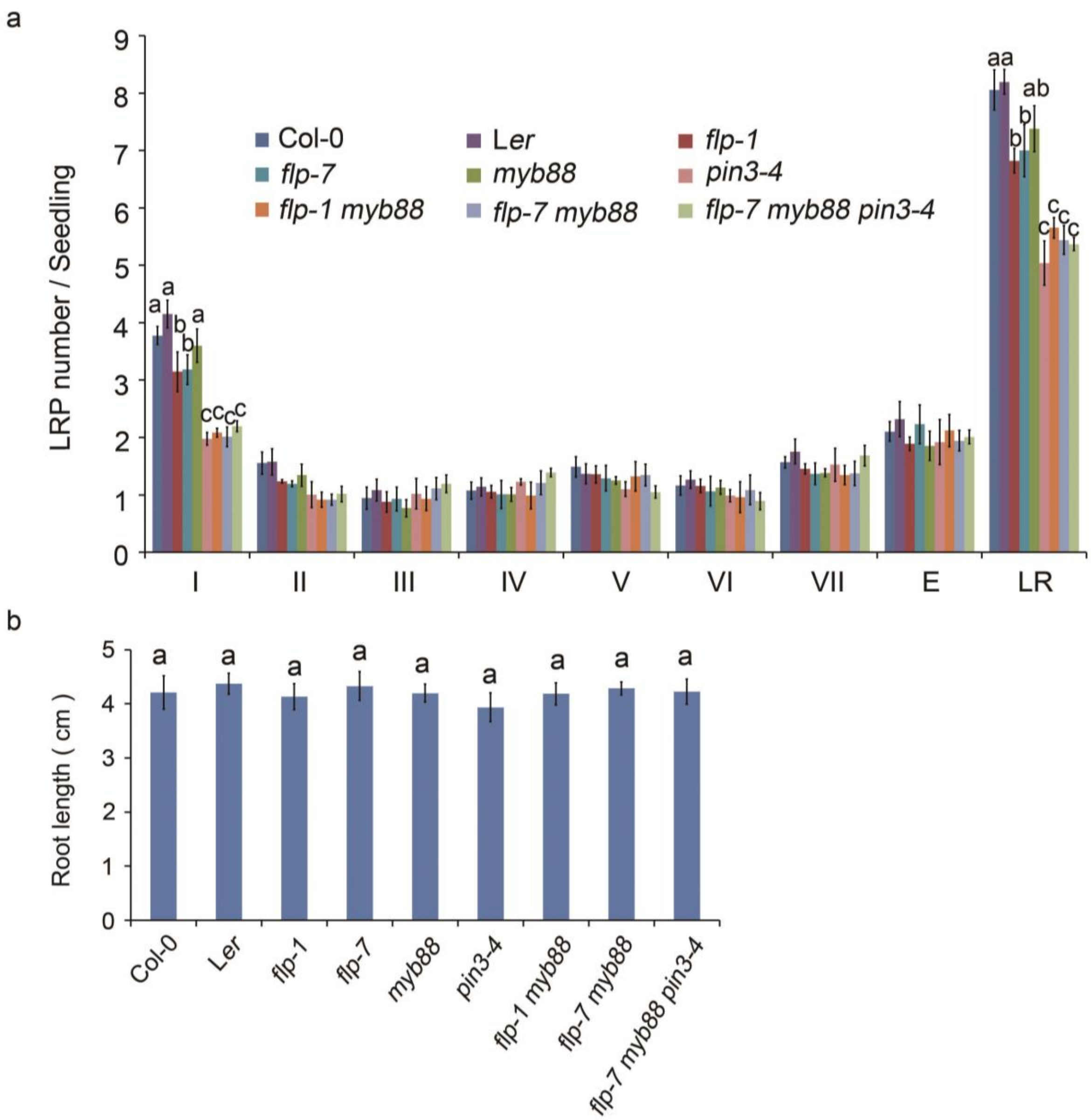

Supplementary Figure 2 Root phenotyping of 6-day-old seedlings of Col-0, L*er*, *flp-1*, *flp-7*, *myb88*, *pin3-4*, *flp-1 myb88*, *flp-7 myb88*, *pin3-4* and *flp-7 myb88 pin3-4*. (**a**) the number of different LR stages per seedling, and (**b)** corresponding main root lengths. E = just emerged, not yet mature LRs; LR = mature LRs. Data corresponding to the densities depicted in Figure 1. Data shown are average and s.d. of at least three independent experiments, each time sampling ($n \geq 20$). Samples with different letters are significantly different: $p < 0.05$ (Fisher's LSD mean separation test).

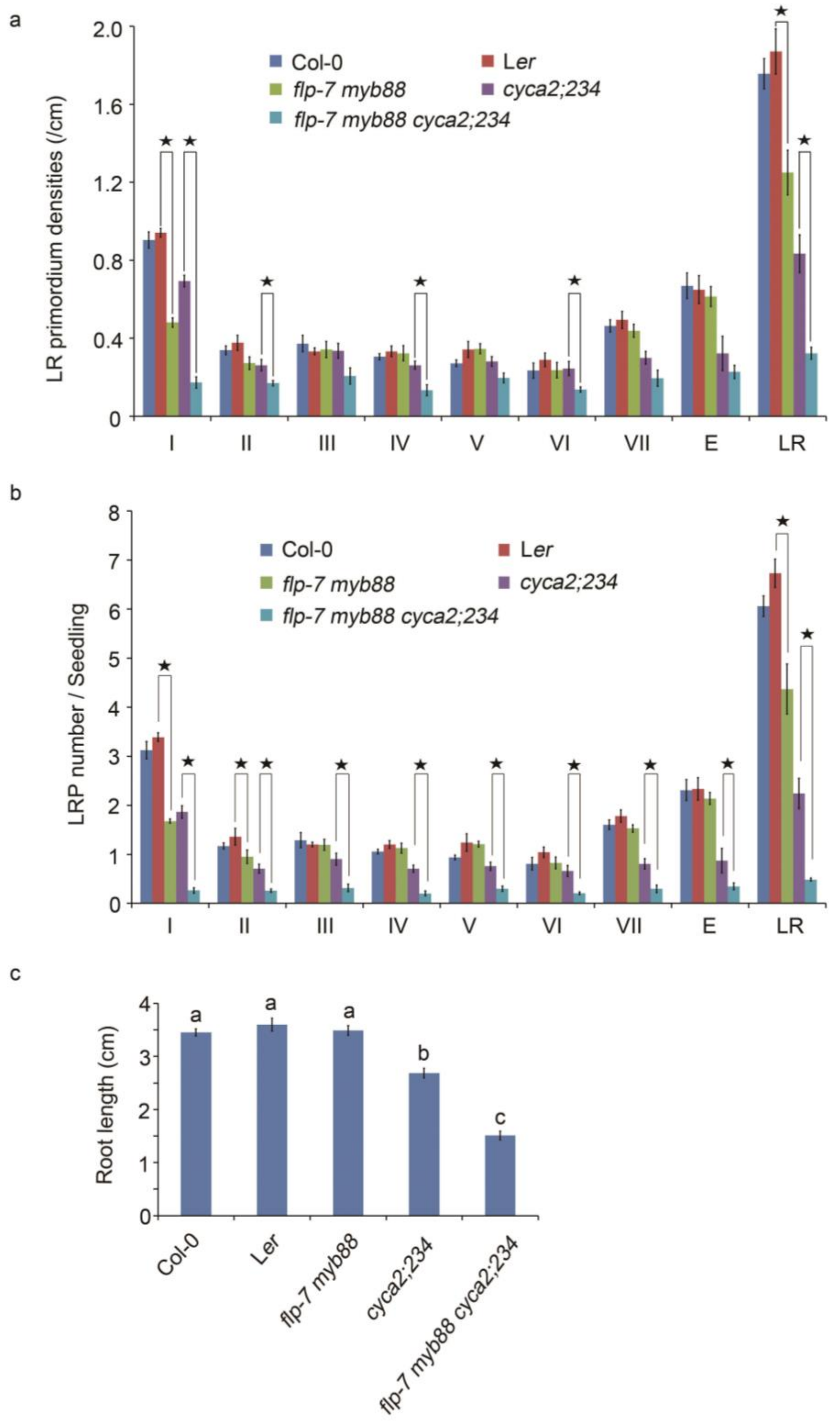

Supplementary Figure 3 Root phenotyping of 6-day-old seedlings of Col-0, L*er*, *flp-7 myb88*, *cyca2;234* and *flp-7 myb88 cyca2;234*. (**a**) LR primordium densities, (**b**) number of LRPs per seedling, and (**c**) primary root lengths of the respective genotypes. E = just emerged, not yet mature LRs; LR = mature LRs. Data shown are average and s.d. and are representative of at least three independent experiments. Asterisks denote Student's *t*-test significance: * $p < 0.001$ ($n > 20$). Samples with different letters are significantly different: $p < 0.05$ (Fisher's LSD mean separation test).

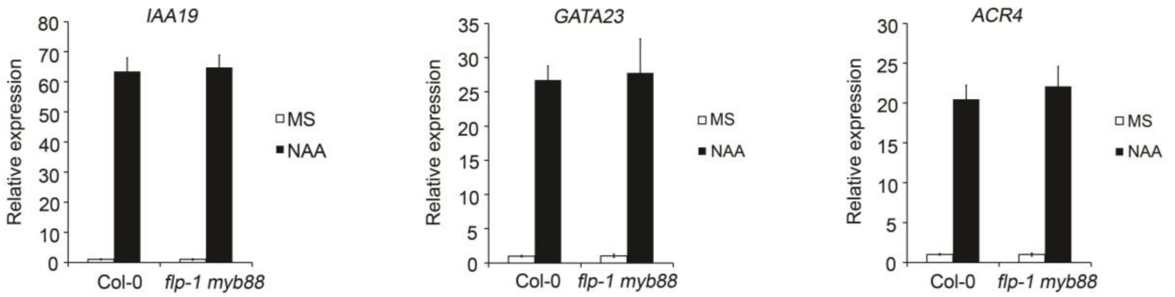

Supplementary Figure 4 Transcriptional response to auxin treatment in WT and *flp-1 myb88* roots for *IAA19*, *GATA23* and *ACR4*. Six-day-old seedlings were treated with 10 μM NAA for 6 hours, and roots were sampled for RNA extraction. Expression levels were normalised to Col-0 (MS). Data shown are average and s.d. and are representative of at least three independent experiments.

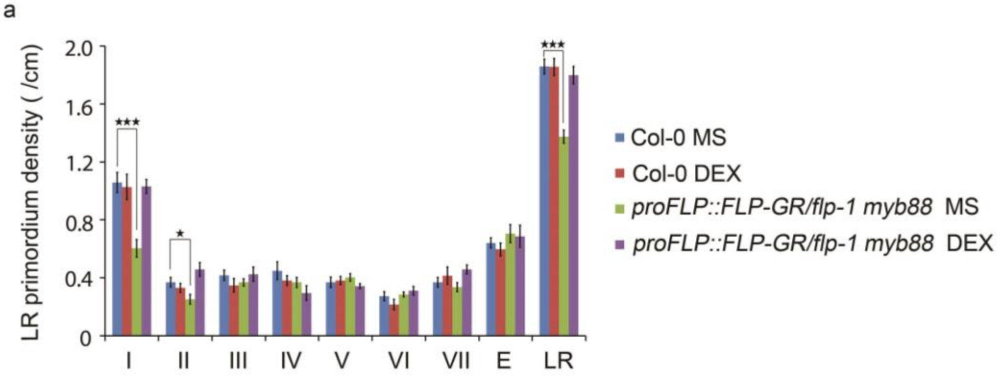

b

Genotype	Cell number in each stomata (%) 2	4	≥6	
Col-0 MS	100	0	0	(n = 269)
Col-0 DEX	100	0	0	(n = 262)
proFLP::FLP-GR flp-1 myb88 MS	37.5	35.3	27.2	(n = 283)
proFLP::FLP-GR flp-1 myb88 DEX	91.9	8.1	0	(n = 332)

Supplementary Figure 5 Phenotypic analyses of *proFLP::FLP-GR/flp-1 myb88* with DEX treatment. (**a**) Densities of the different stages of lateral root development in 6-day-old seedlings of Col-0 and *proFLP::FLP-GR/flp-1 myb88* germinated on MS medium with or without 2 μM DEX. E = just emerged, not yet mature LRs; LR = mature LRs. Data shown are average and s.d. and are representative of at least three independent experiments (n>20). Asterisks denote Student's *t* test significance: * $p < 0.05$ and *** $p < 0.001$. (**b**) Numbers of cells in stomatal complex of Col-0 and *proFLP::FLP-GR/flp-1 myb88* germinated on medium with or without 2 μM DEX for 8 days.

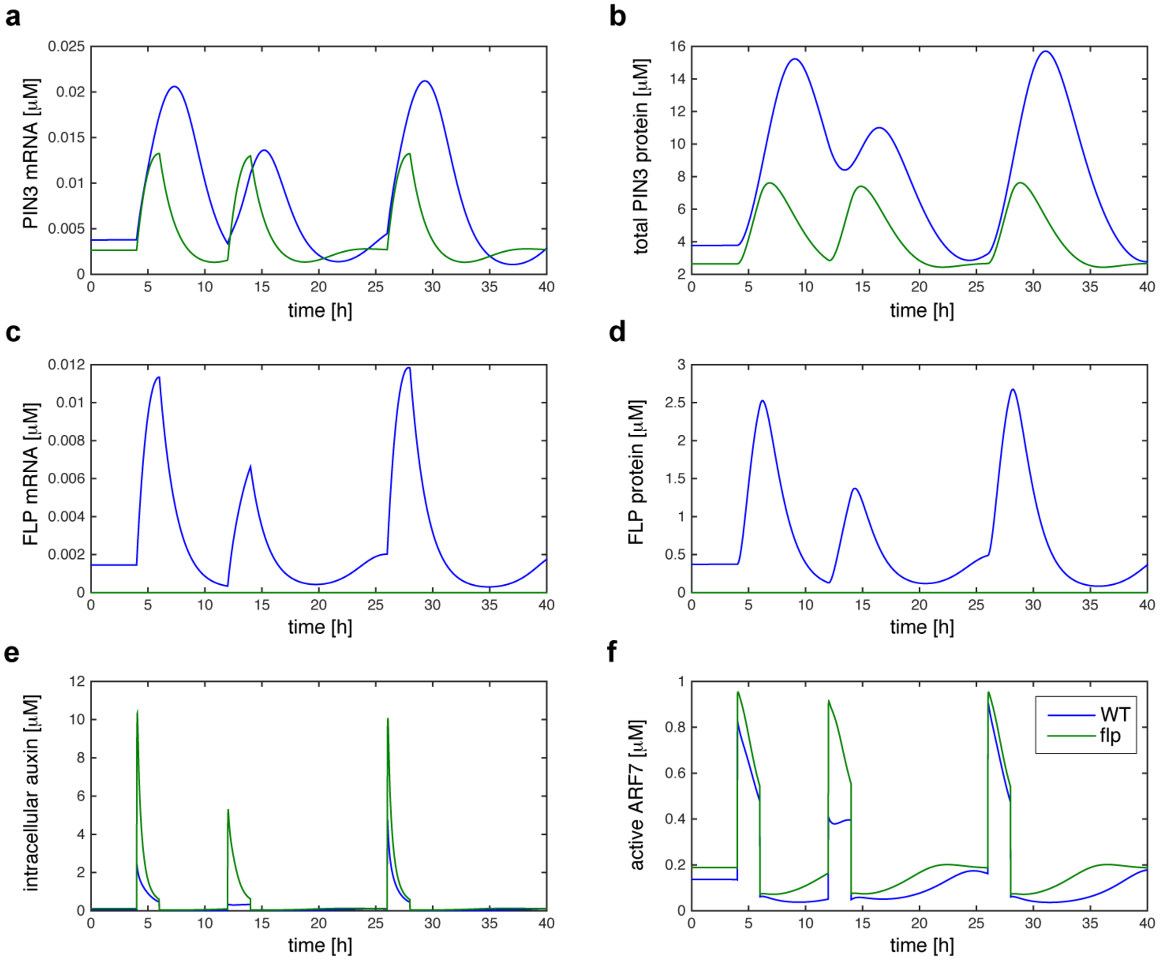

Supplementary Figure 6 Model simulations with (blue) and without (green) the FLP feed-forward circuit. Model parameters are as in Figure 6, except for the half-max constants of the transcriptional circuitry, which were doubled to K_{AF} = 0.6 µM, K_{AP} = 0.6 µM and K_{FP} = 1.2 µM. The resulting system dynamics are qualitatively the same as in Figure 6.

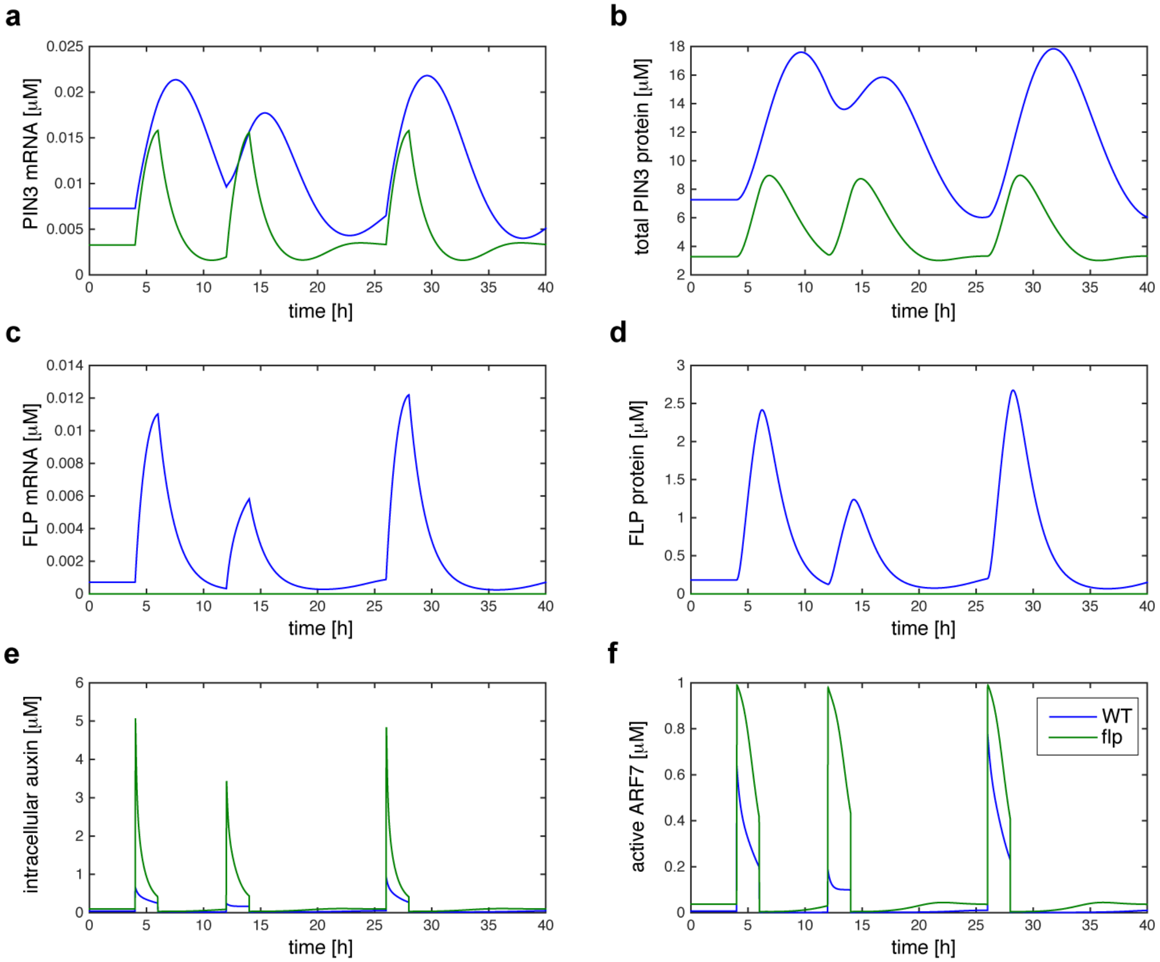

Supplementary Figure 7 Model simulations with (blue) and without (green) the FLP feed-forward circuit. Model parameters are as in Figure 6, except for the Hill coefficients of the transcriptional circuitry, auxin-dependent ARF7 activation and PIN3 membrane localization, which were switched to $n_2 = n_3 = n_4 = 1$ and $n_1 = n_5 = 2$. The resulting system dynamics are qualitatively the same as in Figure 6.

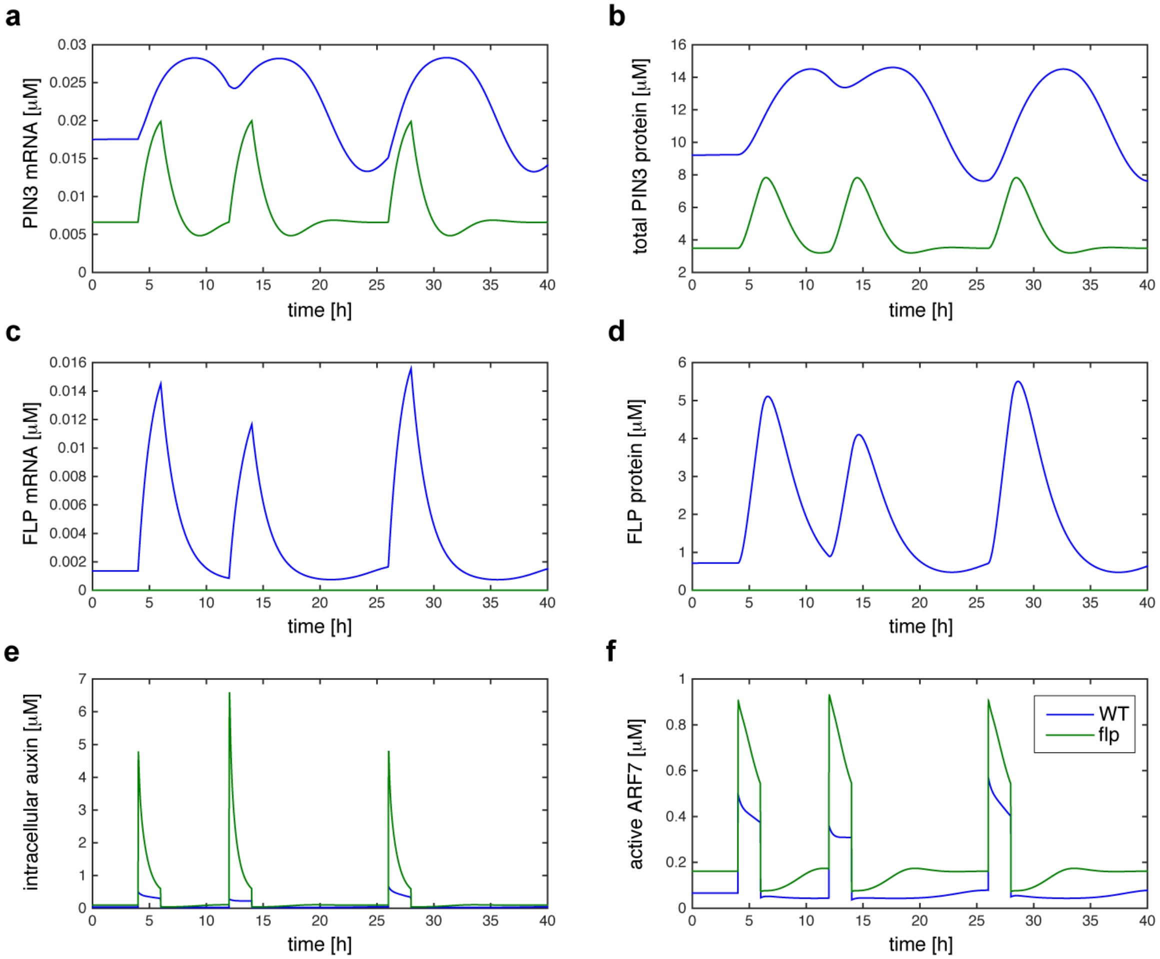

Supplementary Figure 8 Model simulations with (blue) and without (green) the FLP feed-forward circuit. Model parameters are as in Figure 6, except for the PIN3 and FLP protein degradation rates, which were equalized to $\delta_{\mathrm{PIN3}} = \delta_{\mathrm{FLP}} = 1.9 \times 10^{-4}\ \mathrm{s}^{-1}$. The resulting system dynamics are qualitatively the same as in Figure 6.

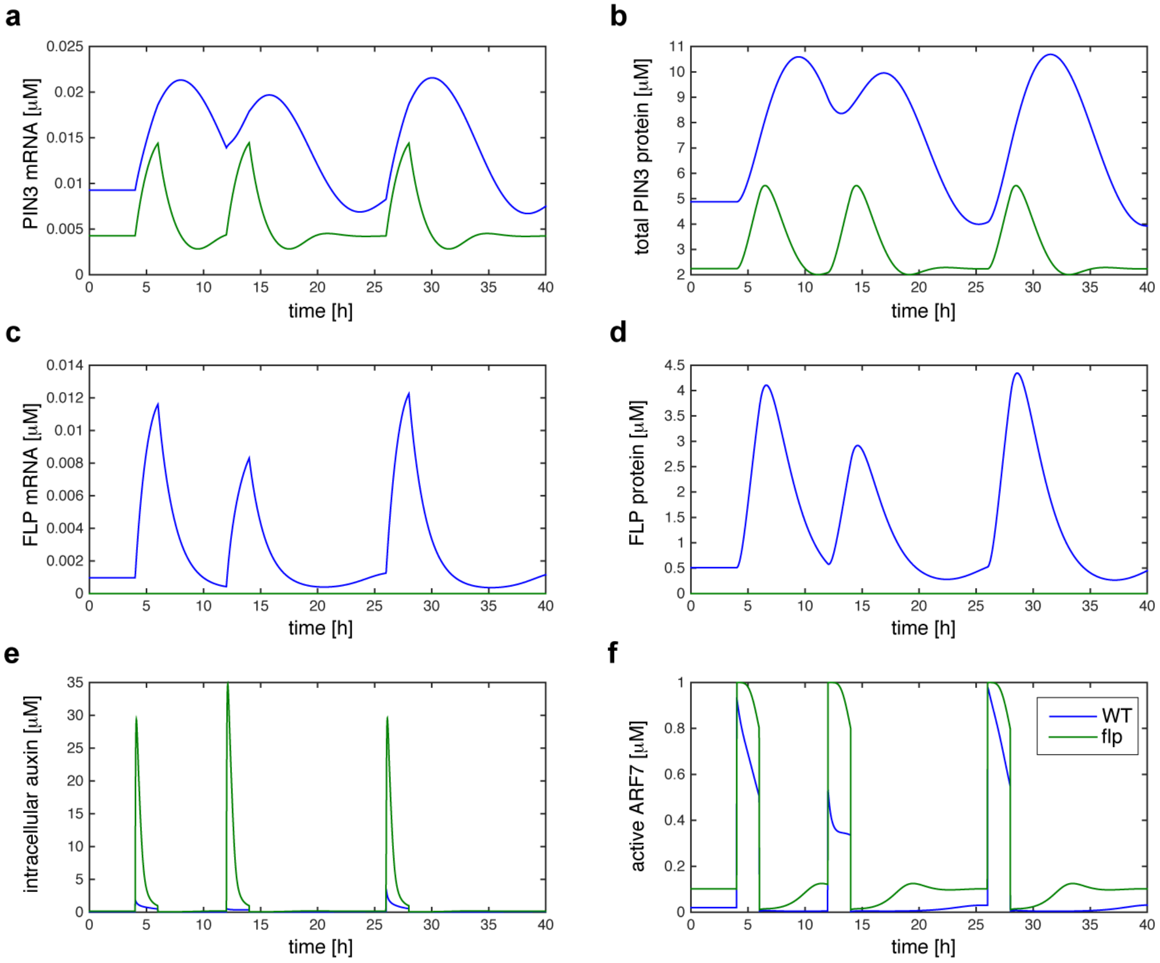

Supplementary Figure 9 Model simulations with (blue) and without (green) the FLP feed-forward circuit, combining the parameter modifications used in Supplementary Figures 6-8. The resulting system dynamics are qualitatively the same as in Figure 6.

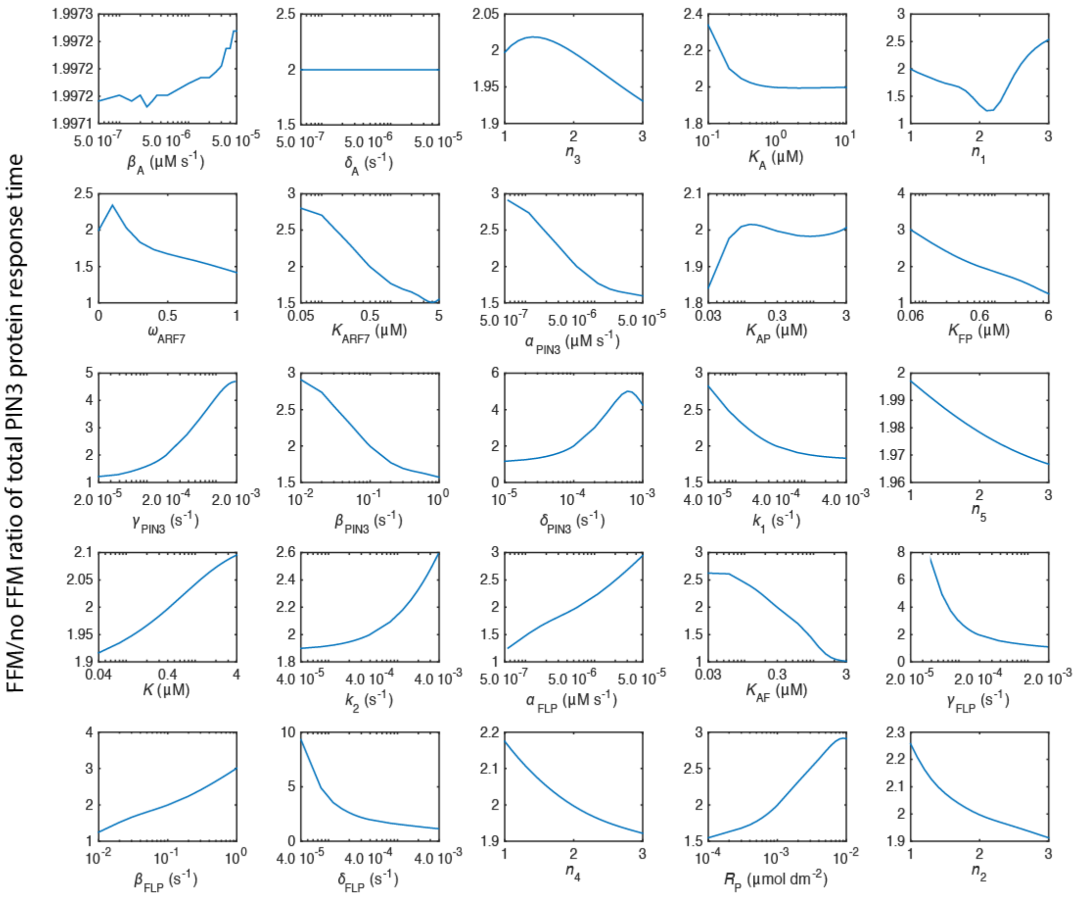

Supplementary Figure 10 Ratio of total PIN3 protein concentration response time for circuits with and without feed-forward motif (FFM) upon auxin stimulus removal, as a function of single parameter deviations from the default values listed in Supplementary Table 1. Ratios >1 indicate that the circuit with feed-forward motif exhibits a delayed response upon stimulus shutdown, i.e. a delayed decrease of PIN3 protein concentration. For none of the parameters, the response time ratio drops below 1 in the parameter range profiled, indicating that the PIN3 response delay is a robust feature of the FLP feed-forward motif circuit. The leftmost data point for γ_{FLP} (γ_{FLP} = 1.9 x 10^{-5} s^{-1}) is missing because the PIN3 protein concentration in the FFM case did not halve within the 40h simulation timeframe.

Supplementary Table 1: Model parameter definitions and default values. Parameter values taken from literature are referenced in the last column.

Parameter	Definition	Default Value	Ref
β_{A}	intracellular auxin production rate	0 μM s^{-1}	
δ_{A}	intracellular auxin degradation rate constant	5 x 10^{-6} s^{-1}	1
$\frac{a}{V}$	cell surface/volume ratio (round cell with 100 μm diameter)	0.06 μm^{-1}	
p_{AH}	membrane permeability AH (auxin acidic form)	3.3 x 10^{1} μm s^{-1}	2
$p_{\mathrm{A^-}}$	membrane permeability A^- (auxin anionic form) at the reference $\mathrm{PIN3_{mem}}$ concentration R_{P}	1.24 x 10^{1} μm s^{-1}	2
R_{P}	reference $\mathrm{PIN3_{mem}}$ concentration	1.0 x 10^{-3} μmol dm^{-2}	
$f_{\mathrm{AH}}^{\mathrm{wall}}$	fraction AH/A in walls	0.334	2
$f_{\mathrm{AH}}^{\mathrm{cell}}$	fraction AH/A in cells	0.003	2
$f_{\mathrm{A^-}}^{\mathrm{wall}}$	fraction A^-/A in walls	0.666	2
$f_{\mathrm{A^-}}^{\mathrm{cell}}$	fraction A^-/A in cells	0.997	2
N_{influx}	electrochemical factor for auxin influx	0.07	2
N_{efflux}	electrochemical factor for auxin efflux	4.0	2
K_{A}	half-max Michaelis-Menten constant for PIN3-mediated transport of anionic auxin	1.0 μM	2, 3
$ARF7$	constant ARF7 protein concentration	1.0 μM	
ω_{ARF7}	basal, auxin-independent ARF7 activity	0	
K_{ARF7}	half-max constant for auxin-dependent ARF7 activation	0.5 μM	
n_1	Hill coefficient for auxin-dependent ARF7 activation	1	
α_{FLP}	maximum *FLP* mRNA synthesis rate	5.6 x 10^{-6} μM s^{-1}	
γ_{FLP}	*FLP* mRNA degradation rate constant	1.9 x 10^{-4} s^{-1}	
K_{AF}	half-max constant for $ARF7_{\mathrm{act}}$-dependent activation of *FLP* transcription	0.3 μM	
n_2	Hill coefficient for $ARF7_{\mathrm{act}}$-dependent activation of *FLP* transcription	2	
β_{FLP}	FLP protein synthesis rate constant	1.0 x 10^{-1} s^{-1}	
δ_{FLP}	FLP protein degradation rate constant	3.9 x 10^{-4} s^{-1}	
α_{PIN3}	maximum *PIN3* mRNA synthesis rate	5.6 x 10^{-6} μM s^{-1}	

γ_{PIN3}	*PIN3* mRNA degradation rate constant	1.9 x 10^{-4} s^{-1}	
K_{AP}	half-max constant for $ARF7_{\mathrm{act}}$-dependent activation of *PIN3* transcription	0.3 μM	
n_3	Hill coefficient for $ARF7_{\mathrm{act}}$-dependent activation of *PIN3* transcription	2	
K_{FP}	half-max constant for FLP-dependent activation of *PIN3* transcription	0.6 μM	
n_4	Hill coefficient for FLP-dependent activation of *PIN3* transcription	2	
β_{PIN3}	PIN3 protein synthesis rate constant	1.0 x 10^{-1} s^{-1}	
δ_{PIN3}	PIN3 protein degradation rate constant	1.0 x 10^{-4} s^{-1}	
k_1	PIN3 membrane localization rate	3.9 x 10^{-4} s^{-1}	
k_2	maximum PIN3 internalization rate	3.9 x 10^{-4} s^{-1}	
K	half-max constant for extracellular auxin effect on PIN3 internalization	0.4 μM	2
n_5	Hill coefficient for PIN3 internalization	1	

Supplementary Table 2: List of primers used

**Cloning**	
proFLP-F	GGGGACAACTTTGTATAGAAAAGTTGGATACATCTACCT ATTTATTGCGCGTAC
proFLP-R	GGGGACTGCTTTTTTGTACAAACTTGTTTTCTTCTTCTTCT TTCTTACTACTGTCTC
FLP-F	GGGGACAAGTTTGTACAAAAAAGCAGGCTTGATGGAAGA TACGAAGAAGAAAAAGAA
FLP-R	GGGGACCACTTTGTACAAGAAAGCTGGGTACAAGCTATG GAGAAGGACTCTT
FLP-R2 (with stop)	GGGGACCACTTTGTACAAGAAAGCTGGGTATTACAAGCT ATGGAGAAGGACTCTT
ARF7-F	GGGGACAAGTTTGTACAAAAAAGCAGGCTTGATGAAAGC TCCTTCATCAAATGGAGTTTC
ARF7-R	GGGGACCACTTTGTACAAGAAAGCTGGGTATCACCGGTT AAACGAAGTGGCTGAGT
proPIN3_FL_F	GGGGACAAGTTTGTACAAAAAAGCAGGCTAGCAACACTA

	AGTCACAAGA
proPIN3_FL_R	GGGGACCACTTTGTACAAGAAAGCTGGGTCTTGAAGGGA CAAAAATGGA
**CHIP-Q-PCR**	
proACT2_F	CGTTTCGCTTTCCTTAGTGTTAGCT
proACT2_R	CACAACGCATGCTAAACAGATCTAG
proPIN3(P1)_F	GAGAATATAGGCTAAAATTTATGTCG
proPIN3(P1)_R	CTAATATCTTGGTACCCCGGCTA
proPIN3(P2)_F	GATAAGATCGATAAAAAATGTAACATA
proPIN3(P2)_R	CTTGAAGGGACAAAAATGGA
proFLP(P1)_F	CGTAACATAATGGCAAGTGTTTTTCTTACA
proFLP(P1)_R	GAACCACACATTTTCTTGATTCAATCTG
proFLP(P2)_F	CGAATCAGAGGAATTATATTGGCAG
proFLP(P2)_R	TTTTCTTCTTCTTCTTTCTTACTACTGT
**Q-RT-PCR**	
PIN1_F	TACTCCGAGACCTTCCAACTACG
PIN1_R	TCCACCGCCACCACTTCC
PIN3_F	GAGGGAGAAGGAAGAAAGGGAAAC
PIN3_R	CTTGGCTTGTAATGTTGGCATCAG
FLP_F	CGAAATGCCACTGGTATTGATAGC
FLP_R	CACCATCACTCTCATTCACATTGC
MYB88_F	GAGGAGATTCACTTTCGGCTTTTAG
MYB88_R	AGGATTGCTTGTTGTGTTAACTCAG
ACR4_F	GATCATAGTGCGGTCTGTTGG
ACR4_R	AGGGATAGAAGCAGGGAAACC
GATA23_F	AGTGAGAATGAAAGAAGAGAAGGG
GATA23_R	GTGGCTGCGAATAATATGAATACC
IAA19_F	GTGGTGACGCTGAGAAGGTT
IAA19_R	CGTGGTCGAAGCTTCCTTAC
ACT2_F	TTGACTACGAGCAGGAGATGG
ACT2_R	ACAAACGAGGGCTGGAACAAG
YFP_F1	ACGGCAGCGTGCAGCTCGCCGACC
YFP_R	CTCCAGCAGGACCATGTGATCGCG

**Yeast-one-hybrid**	
proPIN3FR1_F	AAAGAGCTCAGCAACACTAAGTCACAAGA
proPIN3FR1_R	AAAGTCGACTTAAACTTTTTAACCAAAACAAAAAT
proPIN3FR2_F	AAAGAGCTCACCGATCATCTCTACACTAAATTCA
proPIN3FR2_R	AAAGTCGACATTCTCTAACTAATCCATTTTCGTA
proPIN3FR3_F	AAAGAGCTCGTGAAAGAAAGGTAAAGTAAAATAT
proPIN3FR3_R	AAAGTCGACACATAAATAGTCAAATAATTAAAA
proPIN3FR4_F	AAAGAGCTCGTATGTTGTTATCTACAATATGTCCGTTT
proPIN3FR4_R	AAAGTCGACAAAAAAATAACAATATAGTTCTTTTC
proPIN3FR5 _F	AAAGAGCTCGATAAGATCGATAAAAAATGTAACATA
proPIN3FR5_R	AAAGTCGACCTTGAAGGGACAAAAATGGA
proPIN3_FBS1_F	TGATTATTAGCCGGGGTACCAAGATA
proPIN3_FBS1_R	TCGATATCTTGGTACCCCGGCTAATAATCAAGCT
proPIN3_mFBS1_F	TGATTATTAATTAGGGTACCAAGATA
proPIN3_mFBS1_R	TCGATATCTTGGTACCCTAATTAATAATCAAGCT
proPIN3_FBS2_F	TAGTAATATACCCATATGTTTAATAT
proPIN3_FBS2_R	TCGAATATTAAACATATGGGTATATTACTAAGCT
proPIN3_AuxRE1_F	CCTACTTCACGAGACAAATAAGTAAACC
proPIN3_AuxRE1_R	TCGAGGTTTACTTATTTGTCTCGTGAAGTAGGAGCT
proPIN3_AuxRE2_F	CTTAGTGGATCTTCTTTGTCTCCAGCCCATC
proPIN3_AuxRE2_R	TCGAGATGGGCTGGAGACAAAGAAGATCCACTAAGAGCT
proPIN3_AuxRE3_F	CCCATCTTGTCTCCTTATTTTTCTAATGAT
proPIN3_AuxRE3_R	TCGAATCATTAGAAAAATAAGGAGACAAGATGGGAGCT
proPIN3_mAuxR1_F	CCTACTTCACTCAGCAAATAAGTAAACC
proPIN3_mAuxR1_R	TCGAGGTTTACTTATTTGCTGAGTGAAGTAGGAGCT
proPIN3_mAuxR2_F	CTTAGTGGATCTTCTTCACTTCCAGCCCATC
proPIN3_mAuxR2_R	TCGAGATGGGCTGGAAGTGAAGAAGATCCACTAAGAGCT
proPIN3_mAuxR3_F	CCCATCTCACAACCTTATTTTTCTAATGAT
proPIN3_mAuxR3_R	TCGAATCATTAGAAAAATAAGGTTGTGAGATGGGAGCT
**Promoter Mutagenesis**	
proPIN3_mAuxRE1_F	CTTCACTCAGCAAATAAGTAAACC
proPIN3_mAuxRE1_R	GGTTTACTTATTTGCTGAGTGAAG

proPIN3_mAuxRE2_F	GGATCTTCTTCACTTCCAGCCCATCT
proPIN3_mAuxRE2_R	AGATGGGCTGGAAGTGAAGAAGATCC
proPIN3_mAuxRE3_F	CATCTCACTTCCTTATTTTTCTAATG
proPIN3_mAuxRE3_R	CATTAGAAAAATAAGGAAGTGAGATG
proPIN3_mFBS1_R	GTACCCTAATTAATAATCAAAAAAGCAATAGTT
proPIN3_mFBS1_F	GATTATTAATTAGGGTACCAAGATATTAG

SUPPLEMENTARY REFERENCES

1 Grieneisen, V. A., Xu, J., Maree, A. F., Hogeweg, P. & Scheres, B. Auxin transport is sufficient to generate a maximum and gradient guiding root growth. *Nature* **449**, 1008-1013, (2007).

2 Jönsson, H., Heisler, M. G., Shapiro, B. E., Meyerowitz, E. M. & Mjolsness, E. An auxin-driven polarized transport model for phyllotaxis. *Proc. Natl. Acad. Sci. U. S. A.* **103**, 1633-1638, (2006).

3 Mitchison, G. J. The Dynamics of Auxin Transport. *Proc R Soc Ser B-Bio* **209**, 489-511, (1980).
